# An integrated toolkit for human microglia functional genomics

**DOI:** 10.1186/s13287-024-03700-9

**Published:** 2024-04-10

**Authors:** Imdadul Haq, Jason C. Ngo, Nainika Roy, Richard L. Pan, Nadiya Nawsheen, Rebecca Chiu, Ya Zhang, Masashi Fujita, Rajesh K. Soni, Xuebing Wu, David A. Bennett, Vilas Menon, Marta Olah, Falak Sher

**Affiliations:** 1 Center for Translational and Computational Neuroimmunology, Columbia University Medical Center, New York, NY USA; 2 Taub Institute for Research on Alzheimer’s Disease and Aging Brain, Columbia University Medical Center, New York, NY USA; 3 Department of Neurology, Columbia University Medical Center, New York, NY USA; 4 Department of Medicine, Department of Systems Biology, Columbia University Irving Medical Center, New York, NY 10032 USA; 5 Rush Alzheimer’s Disease Center, Rush University Medical Center, Chicago, IL USA; 6 Proteomics Core, Department of Pathology and Cell Biology, Columbia University Medical Center, New York, NY USA; 7 Neuroimmunology Core, Center for Translational & Computational Neuroimmunology, Division of Neuroimmunology, Department of Neurology, Columbia University Medical Center, New York, NY USA; 8 Department of Physiology and Cellular Biophysics, Columbia University Medical Center, New York, NY USA

**Keywords:** Neurodegenerative diseases, Microglia, iPSC-derived microglia (iMG), Functional genomics, CRISPR, Chromatin accessibility (ATAC-Seq), Proteomics

## Abstract

**Background:**

Microglia, the brain’s resident immune cells, play vital roles in brain development, and disorders like Alzheimer’s disease (AD). Human iPSC-derived microglia (iMG) provide a promising model to study these processes. However, existing iMG generation protocols face challenges, such as prolonged differentiation time, lack of detailed characterization, and limited gene function investigation via CRISPR-Cas9.

**Methods:**

Our integrated toolkit for in-vitro microglia functional genomics optimizes iPSC differentiation into iMG through a streamlined two-step, 20-day process, producing iMG with a normal karyotype. We confirmed the iMG’s authenticity and quality through single-cell RNA sequencing, chromatin accessibility profiles (ATAC-Seq), proteomics and functional tests. The toolkit also incorporates a drug-dependent CRISPR-ON/OFF system for temporally controlled gene expression. Further, we facilitate the use of multi-omic data by providing online searchable platform that compares new iMG profiles to human primary microglia: https://sherlab.shinyapps.io/IPSC-derived-Microglia/.

**Results:**

Our method generates iMG that closely align with human primary microglia in terms of transcriptomic, proteomic, and chromatin accessibility profiles. Functionally, these iMG exhibit Ca2 + transients, cytokine driven migration, immune responses to inflammatory signals, and active phagocytosis of CNS related substrates including synaptosomes, amyloid beta and myelin. Significantly, the toolkit facilitates repeated iMG harvesting, essential for large-scale experiments like CRISPR-Cas9 screens. The standalone ATAC-Seq profiles of our iMG closely resemble primary microglia, positioning them as ideal tools to study AD-associated single nucleotide variants (SNV) especially in the genome regulatory regions.

**Conclusions:**

Our advanced two-step protocol rapidly and efficiently produces authentic iMG. With features like the CRISPR-ON/OFF system and a comprehensive multi-omic data platform, our toolkit equips researchers for robust microglial functional genomic studies. By facilitating detailed SNV investigation and offering a sustainable cell harvest mechanism, the toolkit heralds significant progress in neurodegenerative disease drug research and therapeutic advancement.

**Supplementary Information:**

The online version contains supplementary material available at 10.1186/s13287-024-03700-9.

## Introduction

Microglia, the resident macrophages of the central nervous system (CNS) originate from yolk sac macrophages that populate the developing CNS [1–3]. They play a crucial role in CNS development, homeostasis, and pathobiology of disorders [4] such as Alzheimer disease (AD). While the recent surge of genetic evidence linking microglia to neurodegenerative diseases [5–7] has spurred increased research, functional studies in human microglia remains challenging due to the difficulties associated with obtaining them from human brain and spinal cord autopsy and surgery specimens. Although, immortalized cell lines and mouse models have contributed significantly to our understanding of microglia, genomic instability in continuous cell lines [8] and species-specific variations [9, 10], respectively, represent significant hurdles in translating these findings to humans.

Microglia differentiated from human induced pluripotent stem cells (hiPSCs) [11–15] (iMG) have propelled advancements in the field. However, it is evident that refinements in the differentiation protocols are required, particularly, regarding differentiation time, robustness, efficiency, and most importantly characterization of the generated iMG [16].

Existing protocols [11–13, 17–21] for differentiating iMG typically take over 30 days. Few shorter protocols are reported recently [22] but they require the use of plasmids and antibiotics, which can activate iMG limiting their ability to be manipulated in later experiments. Importantly, in many published protocols iMG characterization is incomplete, lacking information on regulatory DNA and proteome. The information about the regulatory DNA of model iMG is very important for the investigation of functional noncoding sequences implicated in neurodegenerative diseases.

While some protocols [11, 12, 17, 19, 22] offer transcriptomic data through bulk RNA-Seq or expression analysis of microglia makers genes [14], they don’t address heterogeneity among generated iMG. Data accessibility and analysis from these studies also require computational biology skills. Similarly, data on functional attributes of iMG’s (e.g. their phagocytic aptitude, their chemotactic responses, both central characteristic of primary human microglia), is limited. Furthermore, the manipulation of gene expression at large scale, crucial for understanding the molecular role of AD-linked genes, is problematic in iMG generated through published protocols, due to low efficiency and high cell mortality. This current limitation restricts the use of experimental manipulations such as CRISPR-Cas mediated genetic modifications in pooled experiments.

In response to these challenges, this study unveils a toolkit for microglia functional genomics. It comprises an optimized iMG differentiation and culturing protocol, extensive multi-omic validation of the iMG model, a drug-inducible CRISPR-ON/OFF system, and a novel online searchable platform that graphically presents chromatin accessibility (ATAC-Seq), single cell transcriptomic (scRNA-Seq), proteome, and cytokine secretion profiles of newly formed iMG. Notably, the online tool compares the proteome and chromatin accessibility of iMG with primary human microglia, purified from human dorsolateral prefrontal cortex (DLPFC) of the aged brain.

Our toolkit also includes a set of refined functional assays to verify microglial identity. We demonstrate the effectiveness of the Tet-regulated CRISPR-dCas9 system to modify gene expression in iMG, bypassing DNA double-strand breaks through CRISPR-interference (CRISPRi) and CRISPR activation (CRISPRa). Furthermore, we provide detailed step-by-step procedures for human iPSC differentiation into iMG, gene expression manipulation, and assays for assessing the functional and phenotypic consequences of genomic manipulations in iMG.

Our efficient two-step protocol and comprehensive online iMG characterization will facilitate functional human microglia studies, significantly enhancing drug discovery for neurodegenerative diseases.

## Methods

### iPSC culture

Three different human episomal hiPSC cell lines were used for this study. One of the lines was a donation from Columbia Stem Cell Initiative [23], which was derived from dermal fibroblasts from a heathy male. Commercially purchased lines from Gibco (Cat. No. A18945) and ATCC (Cat No. ACS-1024) were derived from the CD34 + cord blood cells from a healthy female and the CD34 + bone marrow cells from a healthy male, respectively. hiPSCs were cultured in mTeSRTM1 (StemCell Technologies, Cat. No. 85,850) media. For regular maintenance, cells were sub-cultured every 3 to 4 days in Geltrex-coated 6-well plates. The growth chamber was maintained at 37°C with 5% CO2. Regular testing for mycoplasma contamination was performed.

### Human astrocyte culture

Normal human astrocytes were sourced from LONZA Bioscience (CC-2565) and maintained in ABM basal medium (Lonza, Cat. No. CC-3187) following LONZA’s guidelines. Upon reaching 80% confluence, cell were transferred to Poly-L-lysine (Sigma, Cat. No. P4707) coated plates for subculturing. For conditioned media production, once astrocytes reached 60% confluence, ABM medium was replaced by microglia progenitor cell medium (composition in supplementary material) and kept for 10 days with media collection every two days. A detailed step-by-step protocol for human astrocyte culture and conditioned medium preparation is available in the supplementary files.

### Two-step differentiation of iPSCs into iMG

A detailed step-by-step protocol for generation of iMG from iPSCs is provided in the supplementary files. In brief, iPSCs were plated in Geltrex coated plates in mTeSR^TM^1 media. In the first step of differentiation, STEMdiff Hematopoietic Kit (StemCell Technologies, Cat. No. 05310) was used for 12 days. The first three days were supplement A was used followed by nine days with supplement B for differentiation. The media was changed every other day. Cells were transferred to Poly-L-lysine (PLL) (Sigma, Cat. No. P4707-50ML) coated plates containing media C^+++^ supplemented with astrocyte-conditioned media (ACM) in a 1:1 ratio. The complete composition of C^+++^ and astrocytic conditioned media is provided in the supplementary step-by-step protocol. Growth factors were added freshly on the day of use. To check the differentiation efficiency, flow cytometry, immunocytochemistry and qPCR were performed to check for marker gene expression after every step. After 8 days in C^+++^-ACM medium, floating cells were collected as microglia-like cells and used for functional or biochemical assays. Cells were collected every 2 to 3 days based on the confluency and plated on PLL coated plates in near homeostatic medium with freshly added growth factors, for functional assays. The composition of near homeostatic medium is provided in the supplementary step-by-step protocol.

### Assay for transposase-accessible chromatin with sequencing (ATAC-Seq)

ATAC-Seq was performed on iMG, and primary human microglia purified from DLPFC of aged brain, using previous describe protocol [24]. The cells were lysed with 50 µl of ATAC-Seq Lysis Buffer (10 mM Tris-Cl, pH 7.4, 10 mM NaCl, 3 mM MgCl_2_, and 0.1% Tween20) and nuclei were plated by centrifugation immediately for 10 min at 500 g at 4 °C. The nuclei pellet was resuspended with 50 µl of transposition reaction mix following the instruction from Nextera Tn5 Transposition Assay Kit (Illumina) and incubated at 37 °C for 30 min. After incubation, DNA was purified using Qiagen MiniElute PCR Purification Kit (Qiagen, Cat. No. 28,104). For library preparation, the purified transposed DNA was first used for partial PCR amplified for 5 cycles with Nextera PCR Primer 1 and Primer 2, using Q5 Hot Start High-Fidelity 2X PCR Master Mix (New England Biolabs, Cat. No. M0494S). A fraction of the PCR product was then used in a side qPCR reaction to determine the appropriate PCR cycle number with minimal amplification biases in the libraries. The PCR amplified libraries were then purified, and double-sided size selected using AMPure XP beads (Beckman, Cat. No. A63881). A Bioanalyzer was used to obtain the size of the purified libraries. The purified libraries were quantified using KAPA Library Quantification Kit (Roche Diagnostics, Cat. No. 50-196-5234) before Next Generation Sequencing (NGS).

### ATAC-Seq analysis

The quality of paired end reads was assessed by FastQC. Alignment of fastq files was performed using Bowtie2 to the human reference genome hg19. Macs2 was used for peak calling with the arguments “–nomodel –nolambda –gsize hs –keep-dup all –min-length 100 –pvalue 1e-5”. Read counts were normalized based on sequencing depth. Consensus peaks among iMG and primary microglia replicates were called with Genrich. Motif enrichment analysis was performed on Identified ATAC-seq peaks using the findMotifsGenome command from HOMER, with parameters “-size 200 -mask.”. overlapping peaks were identified between iMG and primary microglia using the Bedtools intersect function. Known Alzheimer’s disease associated SNPs were identified from the Alzheimer’s Disease Variant Portal [25] and used to call disease variants overlapping microglial accessible regions.

### Single-cell RNA-Seq

#### 10x Genomics chromium next GEM single-cell 3’ library construction

Single cell RNA-seq was performed on iMG using barcoded hashing antibodies [26] staining beta-2-microglobulin and conjugated to hash-tag oligonucleotides (HTOs). For hashing, floating iMG after 20 days of differentiation were collected and counted. Cells were washed with PBS and diluted with cell staining buffer (RPMI containing 1%B-27™ supplement) with added TruStain FcX blocking reagent (BioLegend, Cat. No. 422301). Cells were incubated for 10 minutes at 4°C, then the hash tagging antibody was added to the cells and incubated for additional 30 minutes at 4°C. After incubation, the cells were washed three times with cell staining buffer and recounted for cell viability using disposable counting chambers (Bulldog Bio, Portsmouth, NH). The single-cell library was constructed using 10x Chromium Next GEM Single Cell 3’ Reagent Kits v3.1 (dual Index) with Feature Barcode technology for Cell Surface Protein (10x Genomics, Pleasanton, CA) according to the manufacturer’s protocol. Briefly, a total of ∼ 20,000 cells were loaded on the 10x Genomics chromium controller single-cell instrument. Reverse transcription reagents, barcoded gel beads, and partitioning oil were mixed with the cells to generate single-cell gel beads in emulsions (GEM). After the reverse transcription reaction, the GEMs were broken. Feature Barcode cDNA amplification was performed. The amplified cDNA was then separated by SPRI size selection into cDNA fractions containing mRNA derived cDNA (> 400 bp) and HTO-derived cDNAs (< 180 bp), which were further purified by additional rounds of SPRI selection. The sequencing library was generated from the mRNA and HTO cDNA fractions, which were analyzed and quantified using TapeStation D5000 screening tapes (Agilent, Santa Clara, CA) and Qubit HS DNA quantification kit (ThermoFisher Scientific). The library was sequenced on a NovaSeq 6000 with S4 flow cell (Illumina, San Diego, CA) using paired-end, dual-index sequencing with 28 cycles for read 1, 10 cycles for i7 index, 10 cycles for i5 index, and 90 cycles for read 2.

### Single-cell RNA-Seq data processing and alignment

FASTQ files of the single-cell gene expression library and hashtag oligo library were processed using the “count” command of Cell Ranger (10x Genomics, version 6.0.0) with human transcriptome (GRCh38-2020-A). The default parameters of Cell Ranger were used, except that the max CPU cores were set to 4, and the max memory amount was set to 24 GB. UMIs of HTO library were counted using barcode sequences TGTCTTTCCTGCCAG for control cells with a pattern 5PNNNNNNNNNN(BC)NNNNNNNNN in Read 2.

### scRNA-Seq analysis

10X Genomics Loupe Cell Browser package was used to visualize the cell clustering. An output file (.cloupe) from CellRnager was used as an input for 10X Genomics Loupe Cell Browser package. The .cloupe file contained the expression data for all cells, a t-distribution stochastic neighbor embedding (tSNE), uniform manifold approximating and projection (UMAP) projections and differential gene expression data. The upper UMI threshold was set to minimum 2,000 and maximum 50,000 UMIs per barcode, and the minimum feature count of 100 features per barcode was used for clustering. Cell clusters expressing genes having no significance (*P* ≥ 0.1), calculated by 10X Genomics Loupe Cell Browser with its default algorithm, were removed. Loupe Cell Browser adjusts P values using Benjamini-Hochberg multiple testing correction. The Loupe Cell Browser “Categories” mode was used to label the subpopulations of the cells (clusters) in the clustering plots based on specific categories, while gene/feature expression mode was used to represent expression of a specific gene as a heat map of expression values across all represented cell clusters. The identified clusters were then compared to each other to determine the set of markers genes for each cluster (e.g., genes that were highly expressed in a cluster with respect to all other clusters). The output from Loupe Cell Browser contained list of differentially expressed genes for each cluster with an associated fold change (log_2_ scale) and P values (with Benjamini-Hochberg correction). These statistical values were then exported for further analysis with external software e.g., ShinyGO 0.76.2 and SCANPY packages. The cluster-defining lists of differentially expressed genes are provided in the supplementary data.

### Comparison to the primary microglia scRNA-seq data sets

iMG scRNA-Seq data set was compared to microglial signatures found in two other published datasets. These two data sets were Olah and colleagues [27] (available at Synapse https://www.synapse.org/#!Synapse:syn21438358) and Kracht and colleagues [28] (available at the Gene Expression Omnibus under accession code GSE141862). For the correlation analysis, the unnormalized count values for each gene were summed over all the cells identified as microglia and compared to each other using Pearson correlations after filtering for the intersection of genes detected with our iMG data set and plotted in a correlation plot using Python, with each datapoint showing the gene expression of our iMG data set on the x-axis and the corresponding gene expression of the comparison dataset on the y-axis.

### Stacked violin plots

The filtered matrix, barcode, and features file from the “count” command of Cell Ranger (10x Genomics, version 6.0.0) were then read in the AnnData format using the SCANPY package for further analysis. K-means clustering was done on the cells to cluster them into 4 clusters using the Loupe browser. The cell IDs and the corresponding cluster information were downloaded from the Loupe browser and used to label the cell IDs in the AnnData. iMG were then labeled by filtering for Hashtag 7 labeled cells in the AnnData. Cells with > 3 UMIs were kept for further analysis. The stacked violin plots were visualized using the scanpy.pl.stacked_violin (AnnData) function from the SCANPY package to represent the distribution of RNA counts in each cluster for microglial, monocytic, glial, and neuronal genes.

### Comparison to single-nucleus RNA-Seq from ROS and MAP cohorts

To compare iMG transcriptomic profiles to primary cells in the brain, we integrated the iMG single-cell RNA-seq data with single-nucleus RNA-seq data [29] from post-mortem dorsolateral prefrontal cortex samples from individuals in the ROS and MAP cohorts [30] at Rush University Medical Center. Both studies were approved by an Institutional Review Board and all participants signed informed consent, an Anatomic Gift Act, and a repository consent. After excluding mitochondrial genes, pseudogenes, and antisense transcripts, we ran standard normalization using the SCTransform function in the Seurat R package, followed by dimensionality reduction using 40 PCs, and then integrated the two data sets (iMGs and post-mortem nuclei) using the Harmony package with theta = 4 and lambda = 0.05. UMAP representations for visualization used the first 20 Harmony components.

### Global proteomic profiling of iMG

For global quantitative proteomics of iMG parallel accumulation-serial fragmentation (PASEF) [31] and parallel accumulation-serial fragmentation combined with data-independent acquisition (diaPASEF) [32], two bottom-up proteomics methods, were used.

In brief, 0.4 million cells were lysed in lysis buffer (1% SDC, 100 mM TrisHCl pH 8.5, and protease inhibitors) and boiled for 15 min at 95°C, 1500 rpm. Protein reduction and alkylation of cysteins, was performed with 10 mM TCEP and 40 mM CAA at 45°C for 10 min followed by sonication in a water bath, cooled down to room temperature. Protein digestion was processed for overnight by adding LysC and trypsin in a 1:50 ratio (µg of enzyme to µg of protein) at 37° C and 1400 rpm. Peptides were acidified by adding 1% TFA, vortexed, and subjected to StageTip clean-up via SDB-RPS. Peptides were loaded on one 14-gauge StageTip plugs. Peptides were washed two times with 200 µl 1% TFA 99% ethyl acetate followed 200 µl 0.2% TFA/5%ACN in centrifuge at 3000 rpm, followed by elution with 60 µl of 1% Ammonia, 50% ACN into Eppendorf tubes and dried at 45°C in a SpeedVac centrifuge. Samples were resuspended in 10 of LC buffer (3% ACN/0.1% FA).

### Liquid chromatography with tandem mass spectrometry (LC-MS/MS)

Peptide concentrations were determined using NanoDrop and 200 ng of sample was used for analysis on timsTOFPro. Peptides were separated within 120 min at a flow rate of 400 nl/min on a reversed-phase C18 column with an integrated CaptiveSpray Emitter (25 cm x 75 μm, 1.6 μm, IonOpticks). Mobile phases A and B were with 0.1% formic acid in water and 0.1% formic acid in ACN. The fraction of B was linearly increased from 2 to 23% within 90 min, followed by an increase to 35% within 10 min and a further increase to 80% before re-equilibration.

### diaPASEF

For diaPASEF, the timsTOF Pro was operated in mode1 and data was acquired at defined 32 × 50 Th isolation windows from m/z 100 to 1,700. To adapt the MS1 cycle time in diaPASEF, the repetitions were set to 1.91 in the 32-scan diaPASEF scheme. The collision energy was ramped linearly as a function of the mobility from 59 eV at 1/K0 = 1.6 Vs cm-2 to 20 eV at 1/K0 = 0.6 Vs cm-2. The acquired diaPASEF raw files were searched with the UniProt Human proteome database in the DIA-NN search engine with default settings of the library-free search algorithm 31,768,060. The false discovery rate (FDR) was set to 1% at the peptide precursor and protein level.

### PASEF

For PASEF, the timsTOF Pro was operated in mode1 with the following settings: Mass Range 100 to 1700 m/z, 1/K0 Start 0.6 Vs/cm-2, End 1.6 Vs/cm-2, Ramp time 100 ms, Lock Duty Cycle to 100%, Capillary Voltage 1600 V, Dry Gas 3 l/min, Dry Temp 200 °C, PASEF settings: 10 MS/MS Frames (1.16 s duty cycle), charge range 0–5, active exclusion for 0.4 min, Target intensity 20,000, Intensity threshold 2500, CID collision energy 59 eV. A polygon filter was applied to the m/z and ion mobility plane to select features most likely representing peptide precursors rather than singly charged background ions.

### LC-MS/MS data analysis

Acquired PASEF raw files were analyzed using the MaxQuant environment V.2.1.3.0 and Andromeda for database searches at default settings with a few modifications [33]. The default was used for first search tolerance and main search tolerance (20 ppm and 4.5 ppm, respectively). MaxQuant was set up to search with the reference human proteome database downloaded from UniProt. MaxQuant performed the search trypsin digestion with up to 2 missed cleavages. Peptide, site, and protein false discovery rates (FDR) were all set to 1%. The following modifications were used for protein identification and quantification: Carbamidomethylation of cysteine residues (+ 57.021 Da) was set as static modifications, while the oxidation of methionine residues (+ 15.995 Da), and deamidation (+ 0.984) on asparagine were set as a variable modification. Results obtained from MaxQuant, protein groups table was further used for data analysis.

Results obtained from DIA-NN and MaxQuant, protein groups table were further analyzed using GraphPad Prism. The absolute abundance of each protein was transformed into log_10_. Then based on abundance, all detected proteins were ranked into four quartiles. Finally, abundance of each detected protein was plotted against ranking, using GraphPad Prism. The Venn diagrams were visualized using the matplotlib-venn package by comparing all genes detected in a sample from the mass spectrometry data using python. The protein expression from the mass spectrometry data was plotted in a correlation plot with the RNA expression of our iPSC-derived Microglia data set.

### Functional assays

#### Phagocytosis

##### Phagocytosis of myelin, synaptosome and beta-amyloid

At day 20 of differentiation, floating iMGs were collected and cultured in 8-well chamber slide with near homeostatic iMG medium for three days before performing the independent phagocytosis assays for pHrodo™ iFL Red (ThermoFisher, Cat No. P36014) labeled myelin and synaptosomes and commercially available human recombinant Alexa Fluor 488/647-labeled Aβ1–42 (ANASPEC, Cat No. AS-63327 & AS-65161).

For phagocytosis of Aβ1–42, Aβ was added to a final concentration of 0.5 µM to the cells and incubated for 2 h at 37°C in the growth chamber. Cells were washed three times with PBS and then underwent either for flow cytometric or microscopic analysis using FITC or APC channel. For microscopic analysis, cells were also labeled with IBA1 antibody to identify the cell morphology.

For phagocytosis of synaptosome and myelin assays, 1 µg of pHrodo™ Red conjugated myelin or synaptosome was added to the iMG and incubated for two hours at 37°C inside the growth chamber. After incubation, cells were washed three times with PBS and then underwent either flow cytometric or microscopic analysis using PE channel. For microscopic analysis, the cell morphology was detected by labeling with IBA1 or TMEM119 antibody. Whenever necessary human neuronal (SH-SY5Y) and human microglia (HMC3) cell lines were used as phagocytosis negative and positive controls respectively.

### Extraction of synaptosome from human brain

Synaptosomes were extracted as described in Sellgren, C.M. et al., 2017 [34] with small modifications. Briefly, 200 mg of frozen human dorsolateral prefrontal cortex (DLPFC) tissue was excised from a human postmortem brain specimen. The excised tissue piece was placed on dry ice and minced into small pieces using blade, then immediately transferred to ice-cold Syn-Per buffer (ThermoFisher Scientific, Cat No. 87,793), using 2 ml per 200 mg of tissue, freshly complemented with protease inhibitor cocktail (Millipore Sigma, Cat. No. P8340) and left on ice for 10 min. The tissue was then homogenized using 10 slow strokes of a Dounce homogenizer. The homogenized sample was then centrifuged at 1200 g for 10 min at 4°C, and the supernatant was centrifuged again at 15,000 g for 20 min at 4°C. The synaptosome enriched pellet was resuspended in PBS and quantified using the BCA assay (ThermoFisher Scientific Cat. No. 23,228). Subsequently, freshly purified synaptosomes were conjugated with pHrodo™ according to the manufacturer’s instructions. The enrichment of synaptosomes was confirmed using immunoblotting and anti-SNAP-25 antibody.

#### Extraction of myelin from human brain

For myelin extraction, 2 g of frozen DLPFC human brain tissue was finely sliced on dry ice and transferred to 1.2 ml cold lysis buffer (10 mM HEPES, 5 mM EDTA, 0.3 M sucrose) supplemented with protease inhibitor cocktail. The tissue was further homogenized with 10 slow strokes with a Dounce homogenizer. The tissue lysate was then transferred to ultra-clear tubes for ultracentrifugation. Two layers of sucrose of equal volume; 0.32 M and 0.85 M was added at the bottom of the tube. The sample was then centrifuged at 75,000 g for 30 min at 4 °C. Myelin was collected between the sucrose interface and washed with water at 75,000 g for 15 min. The pellet was resuspended in water and centrifuged at 12,000 g for 10 min at 4°C with maximum acceleration and deceleration. The pellet was resuspended again in water and centrifuged at 75,000 g for 15 min at 4°C. The extraction process was repeated, and the final pellet was resuspended in PBS at 1 mg/ml. Purified myelin was conjugated using pHrodo™ iFL Microscale Protein labeling kit according to the manufacturer’s instructions.

### Measurement of iMG response to inflammatory signals

Floating iMG were collected after 20 days of differentiation and plated on PLL coated 96-well plates or 8-well chamber slides with near homeostatic iMG medium. Cells were grown for three additional days in near homeostatic conditions. Subsequently, cells were stimulated with 1 nM/ml lipopolysaccharide (LPS) (Invitrogen™ 00-4976-03) or vehicle-only for 12 h. Supernatants were collected and cytokine concentrations were measured using human cytokine/chemokine Multiplexing LASER Bead Technology (71-Plex). For the analysis of cytokine/chemokine changes, the fold change of the cytokines/chemokines was calculated by comparing the fluorescent intensities of the cytokines before and after the addition of LPS. We used a two-sided, one-sample t-test on the fold change of the cytokines after the addition of LPS to calculate the p-value for individual cytokines, which was plotted with the fold change of the cytokines in a volcano plot. Finally, the two-sided, paired t-test, paired with individual cytokine/chemokines was used to calculate additional p-value, assessing whether LPS had an overall impact on cytokine/chemokine secretion.

#### Cell migration assay

Floating iMG were collected after 20 days of differentiation for the migration assays. The migration assay was performed using the Calbiochem InnoCyte™ 96-Well Monocyte Cell Migration Assay Kit (EMD Millipore Corp, Cat. No. CBA 098-1REF) following manufacturer protocol. Briefly, 100,000 floating cells were collected and washed with PBS. Cells were resuspended with 100 µl of iMG medium without the supplemented cytokines and placed in the upper chamber of the 96-well assay kit. At the bottom chamber, 150 µl of iMG medium with IL-34 (20 ng/ml) was added. For negative control, iMG medium without IL-34 was used. The THP1 cells were used parallelly to the iMG as positive control of the experiment. The chamber slide was incubated for 12 h in the CO_2_ cell culture incubator. After incubation, medium containing cells from bottom chamber was transferred to a black, conical 96-well plate (provided by the assay kit) and centrifuged for 5 min at 300 g at room temperature. Supernatant was discarded by inverting the plate without dislodging the cell pellet, followed by adding 200 µl of PBS and discarding gently. Cells were resuspended with 100 µl of Cell Labeling Mixture (35 µl of Calcein-AM Solution to 10 ml DPBS) by pipetting up and down several times and incubated for 30 min in the CO_2_ cell culture incubator. Plate was read using TECAN (INFINITE 200 PRO) plate-reader with 465 and 520 nm for excitation and emission wavelength, respectively.

#### ATP evoked intracellular Ca^++^ signaling

Floating iMG were collected after 20 days of differentiation and plated on a 35 mm glass bottom dish with a coverslip (MatTek, Cat. No. NC9341562) for three days in near homeostatic iMG medium. After three days, medium was refreshed containing 2 µM Fluo4 (ThermoFisher Scientific, Cat. No F14201) and incubated for 30 min in the CO_2_ cell culture incubator at 37 °C. The cells were then washed three times with Krebs-Ringer Solution (Alfa Aesar, Cat. No. J67795) containing 2.5 mM probenecid (ThermoFisher Scientific, Cat. No P36400) and were used for microscopy. To record the activity, images were acquired every 5 s on a Zeiss AXIO Observer Z1 microscope using the Zeiss ZEN software in order to record the calcium flux activity of the iMG. The activity was recorded for one minute to acquire the baseline before adding 1 µM ATP (Sigma Aldrich, Cat. No. A2383) or 0.5 mM calcimycin (positive control; Sigma Aldrich, Cat. No. C7522) following by recording for an additional five or ten minutes. The images were processed using CellProfiler software and the Fluo4 intensity values was plotted over time to obtain the activity using Prism software.

#### Flowcytometry

Flow cytometry was performed to confirm the marker gene expression at different stages of differentiation as well as after phagocytosis on fully differentiated iMG. For marker gene expression analysis, 70% confluent iPSCs were detached using Accutase® (ThermoFisher Scientific, Cat. No. A1110501). Differentiating cells were collected at day 12 using Accutase® while iMG were collected as floating cells after 20 days of differentiation. Cells were first washed with PBS and fixed with glutaraldehyde for 10 min at room temperature. After fixing, cells were further washed three times with PBS and resuspended in 0.2 ml of 0.1% Triton X-100 for 5 min at room temperature. 1 ml of 0.3% BSA in PBS (BSA/PBS) was added to the cell suspension and centrifuged for 5 min at 700 g. Cells were resuspended with conjugated antibody in BSA/PBS and incubated for 15 min at room temperature. After incubation, 1 ml BSA/PBS was added, and the sample was centrifuged for 5 min at 700 g. Cells were washed one more time under the same conditions. Finally, cells were resuspended in PBS and analyzed using BD Fortessa analyzer . The details of all used antibody dilutions are provided in the supplementary step-by-step protocol. Data was analyzed using FlowJo software.

### Immunocytochemistry (ICC)

Immunocytochemistry was performed for cell morphology and microglial marker protein expression confirmation. 20,000 floating iMG were plated on a PLL coated 8-well chamber slide and cultured for three days in near homeostatic iMG medium. Once cells were fully differentiated, the media was removed and washed with PBS three times followed by fixing with 4% PFA for 10 min at room temperature. After fixation, cells were washed with PBS three times and blocked with 3% BSA for 30 min. Primary antibody was added in 1% BSA in PBS for two hours at room temperature. Cells were washed three times with PBS and secondary antibody was added for 30 min in 1% BSA in PBS. After secondary antibody incubation, cells were washed with PBS three times and mounted with DAPI (Invitrogen, Cat No. P36931). The details of all used antibody dilutions are provided in the supplementary step-by-step protocol. Images were captured using an Olympus BX3 florescence microscope.

### Drug-inducible CRISPR-ON/OFF expression in iMG

Multiple sgRNAs for CRISPRa (-on) and CRISPRi (-off) were designed using FANTOM and Ensemble data bases to predict transcription start sites (TTS) of target gene [35]. sgRNAs were synthesized by Integrated DNA Technologies (IDT). Annealed sgRNA oligos were ligated into pXPR_502 [36] (CRISPRa) and LentiGuide-Puro [37] (CRISPRi) lentiviral plasmids. After cloning, the sgRNA sequences were verified using Sanger Sequencing. Following sgRNA sequence confirmation, lentiviruses were prepared using LENTI-X™ 293T cells. Simply, LENTI-X™ 293T cells were grown in 15 cm tissue culture treated plates using DMEM medium containing 10% FBS and 1% Penicillin-Streptomycin. LENTI-X™ 293T cells were transfected at 70–80% confluency in 20 ml of media with 13.3 µg of psPAX2, 6.7 µg VSV-G and 20 µg of the lentiviral construct of interest using 180 µg of linear polyethyleneimine and 766.6 ul of OPTI-MEM. Media was changed after 24 h of transfection. Lentiviral supernatant was collected at 72 h of transfection and subsequently concentrated by ultracentrifugation (26,000 RPM for four hours at 4°C with Beckman Coulter SW 32 Ti rotor). Parallel stable hiPSC lines with tetracycline regulatable dCas9-VP64 [38] and constitutive-dCas9-VP64 [35] CRISPRa systems were generated. Similarly, stable hiPSC lines were also generated using tetracycline regulatable dCas9-KRAB [38] and constitutive-dCas9-KRAB [39] expressions.

Once the iPSC lines were stable with either the Tet-dCas9 or the constitutive-dCas9 expression systems, the cells were transduced with sgRNA containing lentivirus. For knock-down and overexpression multiple sgRNAs were tested empirically, and sgRNAs with highest efficiencies were used for downstream experiments. After 48 h of transduction, 1 ng/ml of final concentration of puromycin was added for selection of sgRNA expression. Cells were differentiated following the differentiation protocol as described above and iMG were collected for analysis at day 20. After which the doxycycline was added to final concentration of 0, 0.5, 1.0, 2.0 and 4.0 µM in Tet-dCas-KRAB and Tet-dCas9-VP64 transduced cells. After three days of doxycycline treatment, the iMG were collected and checked for knock-down or overexpression efficiency by qPCR and western blots. Details of knock-down or overexpression are provided in step-by-step protocol in supplementary files.

## Immunoblotting

Cells were harvested and lysed by adding 1X RIPA buffer (Cell Signaling Technology, Cat no. 9806 S) directly to the culturing plate. RIPA buffer was supplemented with freshly added protease inhibitor cocktail and PMSF with 1:1000 dilution from the original concentration. Cells were collected using a cell lifter and transferred to a 1.5 ml Eppendorf tube and placed on ice for 1 h with vortexing every 5 min. The cell lysate was centrifuged at 12,000 g for 5 min at 4°C. The supernatant was collected into a separate pre-chilled tube as protein sample and set on ice. RIPA buffer was added to the remaining cell pellet, vortexed and sonicated for 5 s on/off with 20 amplitudes on ice. Sample was centrifuged with the same condition and the supernatant combined with the previously collected supernatant. Protein concentration was quantified using BCA protein quantification kit using TECAN plate reader.

For western blot, 60 µg of total protein was used for each sample in 4–20% Mini-PROTEAN TGX Precast Protein Gels (Bio-Rad, Cat no 4,561,094). Protein was transferred with semi-wet transfer system using ‘Trans-Blot Turbo Mini 0.2 µm PVDF Transfer Packs’ (BioRad, Cat no. 1704156). The PVDF membrane was blocked for 1 hour with 1:1 ratio of 1X TBS and Intercept (TBS) Blocking Buffer (LICOR, Cat no. 927-60001). After blocking, antibody was added and kept at 4°C for overnight with gentle shaking. The PVDF membrane was washed three times with 1X TBS-Tween® (ThermoFisher Scientific, Cat no. PI28360) after overnight primary incubation. The secondary antibody was added and incubated at room temperature for 1 hour with gentle shaking. The PVDF membrane was washed again three times with TBS-Tween®, and Odyssey LICOR machine was used to obtain the immuno-blot images. ImageJ software was used to further analyze the images. All the antibody concentrations with other details are provided in the extended step-by-step protocol.

### Quantitative real-time PCR

For qPCR, cell harvest and mRNA extraction was performed according to manufacturer protocol (QIAGEN, Cat. No. 74,104). Briefly, 350 µl of RLT Plus buffer with 2 M final concentration of DTT (freshly added) was added and vortexed to make the cell lysate. Cell lysate was transferred to gDNA column and centrifuged at 8000 g for 30 s to remove the genomic DNA. Freshly made 70% ethanol was added to the flow through with 1:1 ratio and transferred to the RNAase column. The sample was centrifuged at 8000 g for 15 s, flow through was discarded and column was washed with RW1 and then with RPE buffer at 8000 g for 15 s. Column was washed with 80% ethanol at 8000 g for 2 min and all extra ethanol residue was removed by centrifuging the column at high speed for 5 min. RNA was eluted in RNase free water.

For cDNA synthesis, 1 µg of RNA was used per reaction in 20 µl reaction (Bio-Rad, Cat no. 1,708,891). For qPCR, amplified cDNA was purified using AMPure XP beads. Subsequently, purified cDNA was quantified using Qubit Fluorometer. 1 ng of purified cDNA was used for 10 µl qPCR reaction. Three technical replicate reactions were performed for each sample. Fast SYBR Green Master Mix (ThermoFisher Scientific, Cat no 4,385,610) was used for qPCR amplification in a 96-well plate using Quant Studio 4.0 (Applied Biosystem). A list of primers is provided in the extended step-by-step protocol.

### Karyotyping

Karyotype analysis of hiPSCs was performed by “Genetica Diagnostic Laboratory” at Columbia University Medical Center. Karyotyping was performed at 70% cell confluency using the standard method followed by the core facility. Briefly, 100 µl of colcemid was added to the cell containing flasks and incubated for 2 h in the CO_2_ cell culture incubator. Media was removed and collected in a 15 ml conical centrifuge tube. Cells were washed with PBS and incubated for 4 min in the CO_2_ cell culture incubator with Collagenase-TrypleExpress (1:1 ratio) mix for cell detachment. Cell suspension was centrifuged at 1200 rpm for 10 min at room temperature, supernatant was discarded and resuspended with 10 ml of 0.54% KCl. The resuspension was incubated for 20 min at 37°C and 10 drops of Carnoy’s fixative was added and then centrifuged for 10 min at 1200 rpm. Cells were washed two more times with freshly added fixative before proceeding to the slide making and G banding.

### Statistical analysis

Experiments were blinded for imaging and data analysis. No sample size calculations were performed, rather, sample sizes were determined to be adequate based on the degree and evenness of measurable differences between groups. Prior literature that has used similar experimental models was also used to choose the sufficient sample sizes. Statistical analyses were performed using GraphPad Prism 9.0 (GraphPad package). For scRNA-Seq analysis P values were adjusted using Benjamini-Hochberg correction for multiple tests. Two-sided one-sample t-test, and two-sided paired t-test paired with individual cytokine/chemokine were used to test the effect of LPS on cytokine secretion. Means between two groups (qPCR analysis) were compared using two-tailed paired/unpaired Student’s t-tests. Detailed information on sample sizes, number of replicates and statistical tests used from each experiment are available in figure legends.

## Results

### Refining human iPSC differentiation into iMG

Leveraging previously published protocols [12–14, 19] for generating iMG from human iPSCs (hiPSCs), we have developed a two-step method which reduces differentiation time and improves iMG purity and survival and enables their in-depth characterization. We utilized three hiPSC lines: a previously published male line [23] (CU-iPSC) from The Stem Cell Core Facility at Columbia University, a commercially procured line from male bone marrow CD34^+^ cells using Sendai virus (C2-iPSC), and a commercially available human episomal iPSC line (C1-iPSC), derived with zero footprint from the CD34^+^ cord blood cells of a female individual using an EBNA-based episomal system [40, 41].

**Fig. 1:**
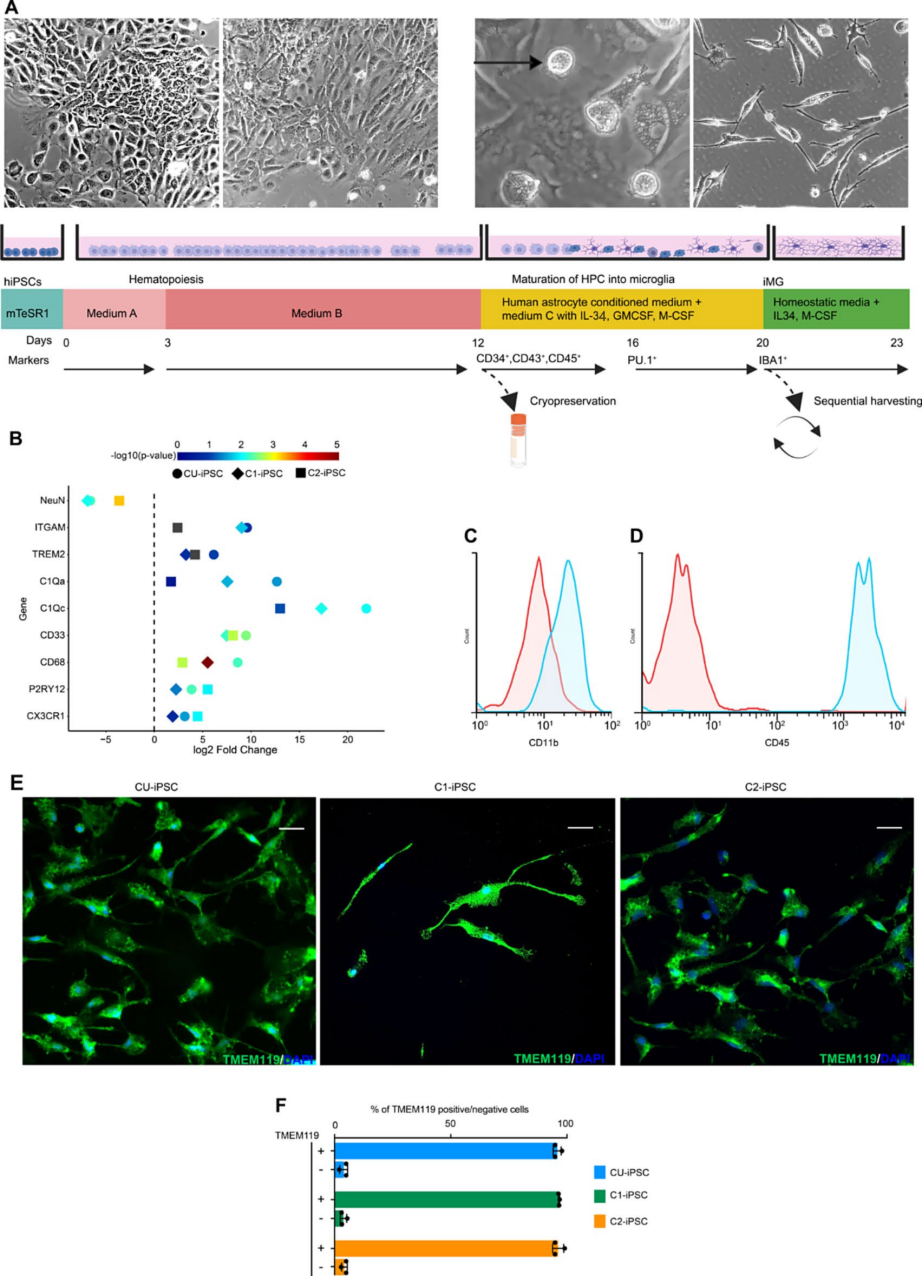
Differentiation of hiPSCs into microglia-like cells (iMG). **A**. Schematic overview of two-step differentiation of hiPSCs into iMG. In the first step the hiPSCs are differentiated into hematopoietic progenitors (HPCs) using media A (days 0–3), followed by media B (days 3–12) from a commercial kit. At day 12, cells exhibit CD34^+^, CD43^+^, and CD45^+^ expression and are transferred to poly-L-lysine coated plates containing media C^+++^ supplemented with astrocyte-conditioned media. (HPCs can be cryopreserved at this level). By day 16, PU.1 transcription factor emerges, and non-adherent round IBA1/TMEM119^+^ iMG appear on day 20. IBA1/TMEM119^+^ iMG can be sequentially harvested up to six times and upon further 2–5 days of culturing in defined media, they acquire homeostatic properties. The upper panel shows the representative images of differentiating iPSCs, HPCs, floating iMG (black arrow), and iMG cultured in near homeostatic conditions. **B**. qPCR data shows the expression of microglial signature genes in iMG derived from three different iPSCs lines. Color and style denotes p-values and iPSC line, respectively, with the X-axis displaying the log2 fold change. **C**-**D**. Flow cytometry analysis shows the expression of CD11B and CD45 on day 20 iMG. Red histograms represent the isotype control staining, blue histograms show the CD11B and CD45 staining. **E**. Immunocytochemistry (ICC) of TEMEM119 expression under near homeostatic conditions in iMG derived from indicated iPSC lines. Scale bars represent 10 μm. **F**. Displays relative percentage of TMEM119 positive and TMEM119 negative cells in iMG cultures differentiated from three independent iPSC lines

In the first stage, we differentiated hiPSCs into hematopoietic progenitor cells (HPCs) using a commercial hematopoietic differentiation kit (STEMdiff). In the second stage, HPCs differentiated into microglia like cells in a mix of human astrocyte conditioned media and an enriched media (C^+++^) adapted from Pandya, H. et al. [13] and Andreone et al. [14] (IMDM, defined FBS, IL-34, GM-CSF, and M-CSF). The entire process took 20 days – 12 days for hematopoietic differentiation followed by 8 days of microglial differentiation (Fig. 1A).

Unlike Andreone et al., we directly cultured HPCs on poly-D-lysin coated plates, in a 1:1 mix of normal human astrocyte conditioned media and C^+++^ media while maintaining the final concentrations of defined FBS at 10% and IL-34, GM-CSF, and M-CSF to 20 ng/ml each. Earlier studies [13, 14] that cocultured HPCs with proliferating normal human astrocytes, led to the presence of GFAP positive cells in the resulting iMG (Figure S1A-B). We achieved hematopoiesis (step 1) by culturing dissociated hiPSCs in STEMdiff kit per the manufacturer’s instructions e.g., 3 days in medium A followed by 8 days in medium B (Fig. 1A). On day 12, HPCs exhibited CD34 and CD43 expression (Figure S1C) and were transferred to human astrocyte conditioned media supplemented with C^+++^ media for microglia maturation (step 2, Fig. 1A). In 3–4 days, cells started to express microglial transcription factor PU.1 and IRF8 (Figure S1D and extended data Figure Ext. 1). Seven days later, we harvested mature microglia-like floating cells (iMG) from all three iPSC differentiating lines. Concurrently, the attached progenitor cells continued to proliferate, enabling us to harvest iMG at least six times in succession before depleting the progenitor cultures, suggesting our differentiation strategy is compatible with high throughput assays such as pooled CRISPR screens. Starting with 50,000 iPSCs in one well of 12-well plate, yielded sufficient HPCs for cryopreservation and produced 1.2 million iMG using only two wells of a six-plate for HPCs differentiation into iMG (Figure S1E) Verification of iMG identify across all iPSC lines was established by consistent expression of microglia signature genes such as *CX3CR1*, *P2RY12*, *CD68*, *CD33*, *TREM2*, *C1QA*, *C1QC, ITGAM* (Fig. 1B) and by iMG expression of microglial marker proteins CD11b and CD45 (Fig. 1C-D).

Next, we incorporated the use of a near homeostatic media from Muffat. et al. [12]. Freshly harvested iMG, seeded on this media on poly-L-lysine coated surfaces, showed IBA1/TMEM119 immunoreactivity across all tested iPSC lines (Fig. 1E and extended data Figure Ext.2). Using filtered astrocytic conditioned media resulted in more than 95% TMEM119 positive iMG yield (Fig. 1F). This level of purity suggests that an additional purification step may not be necessary for most applications. iMG in near homeostatic media exhibited characteristic morphology of in vitro cultured human primary microglia (round or spindle with processes), as described in previous studies [42]. When categorizing the iMG as round or spindle-shaped, the distributions among the iPSC lines used were as follows: CU-iPSC at 57:43%, C1-iPSC at 44:56% and C2-iPSCs at 68:32% (Figure S1F).

Furthering our experiments, we determined that the prime time for cryopreserving of differentiating iPSCs, is on day 12, when they have transitioned into HPCs. By cryopreserving at the HPCs stage, we could reduce the total differentiation duration to just 8 days. The benchmarks outlining our iMG differentiation and characterization are presented in Table 1.

A comprehensive step-by-step refined protocol for hiPSCs differentiation into iMG is provided in Supplementary material.

**Table 1:** iMG differentiation and characterization benchmarks (MS: mass spectrometry, ATAC-Seq: assay for transposase-accessible chromatin with sequencing, sc: single cell, hSNS: human synaptosome, mNeuron: mouse neuron, hAstro: human astrocytes

iMG characterization	New protocol	Muffat et al., [12]	Abud et al., [11]	Douvaras et al., [17]	Haenseler et al., [18]	Pandya et al., [13]	Takara et al., [20]	McQuade et al., [19]	Andreone et al., [14]	Drager et al., [22]
Days required for Differentiation	20	74	38	65	70	29	46	38	23	8
Step-by-step protocol reported	Yes	No	No	No	No	No	No	No	No	No
Multi-omic web-resource	Yes	No	No	No	No	No	No	No	No	No
Sequential harvesting of iMG	Yes	No	No	No	No	No	No	No	Yes	No
Proteomics profiling of iMG	MS-global Proteomics	No	No	No	No	No	No	No	No	No
Chromatin accessibility profiling of iMG	Yes, ATAC-Seq	No	No	No	No	No	No	No	No	No
Transcriptional profiling of iMG	scRNA-seq	RNA-seq	RNA-seq	RNA-seq	None	Microarray	None	RNA-seq	RNA-seq	RNA-seq
Cytokine expression profiling	LASER-bead 71plex	Cytokine Array Kit	V-PLEX 30-plex	Cytokine Array Kit	Luminex100 Bio-Plex	TNF-α	Magnetic Luminex	None	mRNA expression	36-cytokine proteome
No. of plasmids used for iMG differentiation	0	0	0	0	0	0	0	0	0	2
Phagocytosis Assays	Aβ, hSNS, myelin	Latex beads	*E coli*, hSNS	Latex beads	Zymosan	*E coli*	Beads, AMRA- Aβ	Aβ, S.a*ureus*, zymosan	mNeuron/myelin	Rat-SNS, beads
Assessment of iMG calcium signaling	Calcimycin, ATP-response	None	ADP-response	ADP-response	None	None	None	None	None	None
iMG culture system	Mono-culture	Mono-culture	Mono-culture	Mono-culture	Mono-culture	hAstro/iMG co-culture	Neuron/iMGco-culture	Mono-culture	hAstro/iMGco-culture	Mono-culture
Antibiotics required for iMG differentiation	None	None	None	None	None	None	None	None	None	Doxycycline
Homeostatic culturing conditions reported	Yes	Yes	No	No	No	No	No	No	Yes	No

### Chromatin accessibility profiling identifies iMG’s regulatory resemblance to human primary microglia

To investigate if our iMG is a viable model for studying functional noncoding sequences related to neurodegenerative diseases like AD, we performed global open chromatin profiling using the assay for transposase-accessible chromatin with sequencing (ATAC-Seq) [43]. We compared chromatin profiling of iMG to primary microglia (pMG) purified from aged human dorsolateral prefrontal cortex, using an established protocol [27, 44].

Before constructing ATAC-Seq libraries, we verified the expression of microglia signature genes (*AIF1, TMEM119, P2RY12, CD68, TREM2, C1QA, C1QC, ITGAM)* in iMG. Subsequent sequencing yielded an average of over 150 million mappable reads per sample. The quality assessment of ATAC-Seq libraries, compared against prior ATAC-Seq data, revealed high data quality, supported by sensitivity analysis and nucleosome periodicity (Figure S2A-C).

**Fig. 2:**
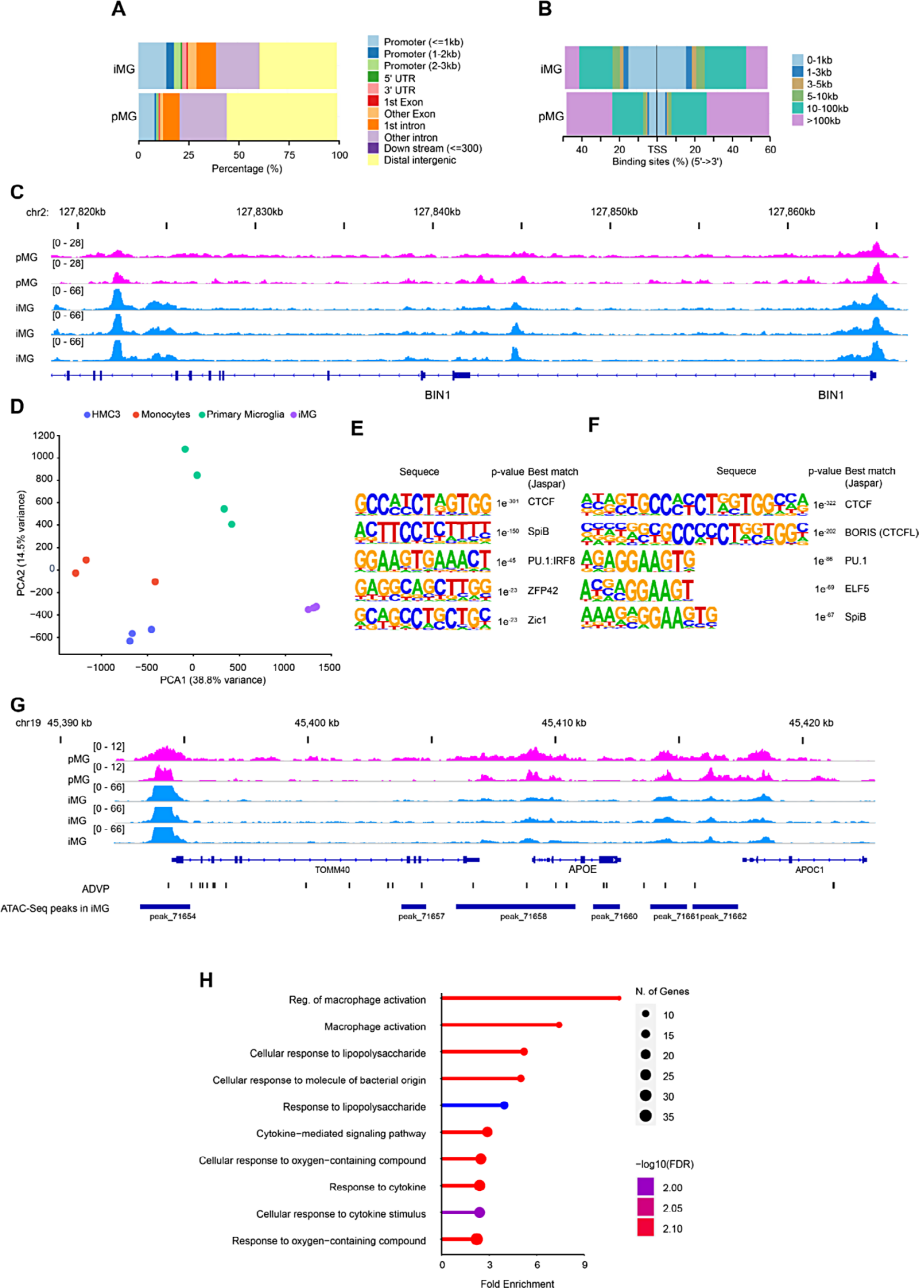
Mapping open chromatin by ATAC-Seq in iMG and human primary microglia. **A**. Displays distribution of DNA accessible sites (DAS) across each genomic feature in human iMG and primary microglia (pMG). **B**. Illustrates the relative distance of DAS from transcriptional start sites human iMG and primary microglia (pMG). **C**. ATAC-Seq abundance tracks identify accessible chromatin regions (DAS) around the BIN1 gene in iMG (blue tracks) and pMG (pink tracks). **D**. Principal component analysis (PCA) of ATAC-Seq signal at a microglia specific gene set (see Table S1). Data sets include iMG, pMG and monocytes and HMC3 cells from another study in our laboratory. **E**-**F**. De novo (**E**) and known (**F**) motif discovery analysis (HOMER) on common DAS in iMG and pMG are presented. **G**. AD-linked APOE variants concentrate on genomic regulatory regions (DAS) in iMG and pMG. ADVP track shows the AD-associated single nucleotide variants around APOE from ADVP database. DAS detected in the vicinity of APOE gene in iMG are shown as blue dashes. **H**. Gene ontology (GO) enrichment analysis for 250 genes associated with most significant common peaks between iMG and pMG is depicted. ATAC-Seq was performed on iMG derived from C1-iPSC line. Abbreviations: UTR untranslated region; TSS transcription start site; kb kilobase; HMC3 human microglia cell line 3

The ATAC-Seq data processed using ENCODE pipeline revealed 159,995 DNA accessible sites (DAS) in iMG and 141,523 in pMG. These sites were found across various genomic features in both cell types (Fig. 2A). Consistent enrichment of DAS around the transcription start site confirmed data integrity and replicability (Fig. 2B). Individual loci, for example BIN1 an AD-linked gene, showcased DAS presence in both iMG and pMG, especially in promoter and intergenic regions (Fig. 2C). Simultaneously, there were no DAS observed around the pluripotent stem cell markers (NANOG, POU5F1 (OCT4)) astrocyte marker (GFAP), neuron marker (RBFOX3), or oligodendrocyte marker (MOG)(Figure S2D), indicating the complete differentiation of iPSCs into iMG and confirming their identity and purity.

Next, we conducted principal component analysis (PCA) on DAS around microglial signature genes and AD genes (Table S1) comparing iMG to pMG, monocytes and human microglia clone 3 (HMC3) cell line. Interestingly, PCA revealed striking similarity between iMG and pMG (Fig. 2D). Using the HOMER algorithm on shared peaks between iMG and pMG, we identified top de novo motifs including CTCF, SpiB, PU.1, IRF8, ZFP42, and Zic1 (Fig. 2E) and top known motifs like CTCF, CTCFL, PU.1, ELF5 and SpiB (Fig. 2F).

Additionally, when aligning AD-associated variants from the Alzheimer’s Disease Variant Portal (ADVP) [25] to DAS in both cell types, most variants were located within DAS in iMG, mirroring those in pMG, as illustrated in Fig. 2G. This suggests that our iMG can serve as model system to investigate non-coding AD-associated genetic variants found in genomic regulatory regions.

Finally, gene ontology analysis (GO) of top (250) shared DASs between iMG and pMG spotlighted terms related to immune cell function (Fig. 2H), emphasizing the immune characteristics shared between iMG and pMG.

In summary, iMG’s DAS show a strong resemblance to those in human pMG.

Detailed comparison of DAS between iMG and pMG is accessible through our online portal https://sherlab.shinyapps.io/IPSC-derived-Microglia/.

#### Single-cell transcriptome analysis of iMG

To systematically understand identity and phenotypic heterogeneity of our iMG cultures, we performed single-cell gene expression analysis by using 10x Genomics platform for single-cell RNA sequencing (scRNA-Seq). By day 20 of differentiation, iMG were harvested and processed for scRNA-Seq (see Figure S3A and Methods). Approximately 2000 cells passed quality control and served as our final dataset.

**Fig. 3:**
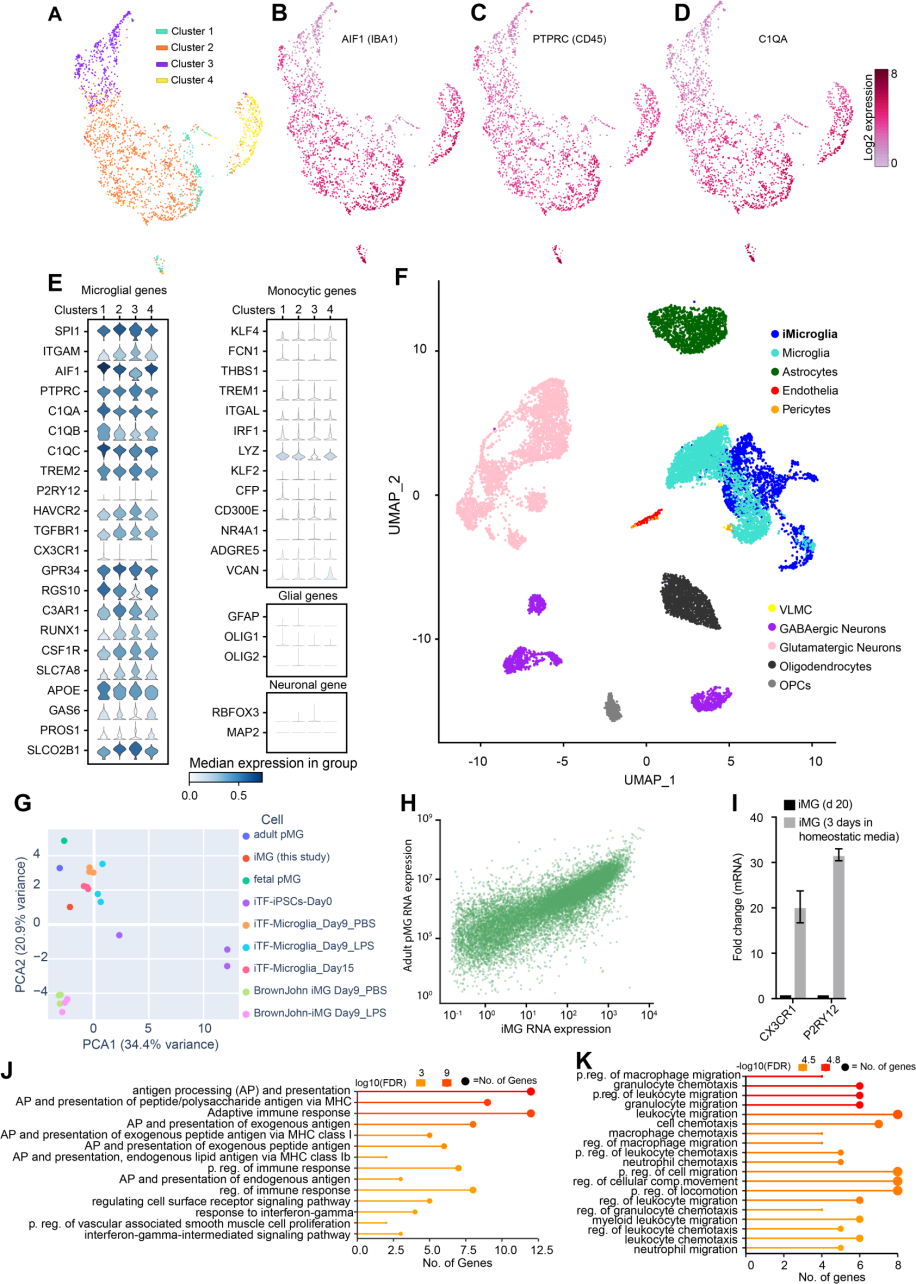
Single-cell gene expression analysis of iMG. **A**. K-means clustering of ~ 2000 single-cell transcriptional profiles from iMG at day 20, identifying four transcriptionally distinct clusters. These iMG clusters are presented in a Uniform Manifold Approximation and Projection (UMAP), where each dot represents a cell. The cells are color-coded based on their cluster affiliation. **B**-**D**. The expression of microglial genes *IBA1*, *CD45*, and *C1QA* projected on the UMAP representation of the clusters. The colored scale represents the log2 expression. **E**. Stacked violin plots of ~ 2000 iMG showing cluster-wise the high expression levels of microglial genes (left panel) compared to the low or absent expression of monocytic genes (right upper panel). The expression of glial and neuronal markers (e.g., astrocytes (*GFAP*), oligodendrocytes (*OLIG1* and *OLIG2*) and neurons (*RBFOX3* and *MAP2*)) was not detected (right lower panels). **F**. UMAP showing the integration of our iMG-scRNA-Seq data with the post-mortem single-nucleus RNA-Seq data from aging human frontal cortex (Cain, A. et al. 2023). The integration revealed that iMG signature resembles the microglial signature but differs from the signature of other cell types in the brain, including neurons, neuroglia, and vascular cells. **G**. Principal component analysis of expression of microglial genes in iMG (red) human adult microglia (blue), human fetal microglia (green) and iPSC-derive iMG from other studies, showing strong resemblance of our iMG to primary microglia. **H.** Scatter plot showing the comparison of iMG mRNA expression with that of primary adult aged microglia (pMG) from a previous study, which were purified from the dorsolateral prefrontal cortex of deceased patients (Olah, M. et al. 2020). Each dot represents a gene. A strong correlation between the transcriptome of iMG and pMG was observed (Kendall’s tau correlation coefficient (T_b_) = 0.6826, p-value = 0.0, Pearson’s *r* = 0.2658 and p-value = 8.771e^− 288^). **I**. Bar graph comparing *CX3CR1* and *P2RY12* expression in iMG differentiated for 20 days and iMG cultured in homeostatic conditions for additional three days (total 23 days). Fold changes were identified by quantitative RT-PCR (*n* = 3, C1 iPSCs). Error bars represent the standard error of three independent experiments. **J**-**K**. Gene enrichment analysis of the top 25 upregulated genes in each cluster. Lollipop charts provide information about Gene Ontology (GO) fold enrichment, significance (FDR log10), and the number of genes in each enriched term in iMG cluster 1 (**J**) and cluster 2 (**K**). scRNA-Seq was performed on iMG derived from C1-iPSC line. Abbreviations: Uniform Manifold Approximation and Projection (UMAP); PCA principal component analysis; VLMC vascular leptomeningeal cell; OPC oligodendrocyte precursor cells; FDR false discovery rate

Cell Ranger and Loupe Browser-based clustering and visualization with uniform manifold approximation and projection (UMAP) identified four distinct cell populations (Fig. 3A). Notably, all four populations (clusters) showed strong expression of microglia marker genes (Fig. 3B-D). Cluster 1 displayed increased levels of high-affinity receptor for immunoglobulin E (*FCER1A*) and human leukocyte antigen (HLA) complex genes (e.g., *HLA-DQB1, HLA-DPA1, HLA-DPB1, HLA-DRB1, HLA-DQA1*, and *HLA-DRA*), whereas genes involved in macrophage/microglial migration, chemotaxis (e.g., *CXCL8, HBEGF, CREM, IL1B, C5AR1*) and immunoglobulin Fc receptors (*FCGR2A*) were prevalent in Cluster 2 (Figure S3B), our largest cluster representing 58% of the total cells (Fig. 3A). The relative fold difference of marker genes in Cluster 2 was smaller, suggesting that these genes displayed strong expressions also in other three clusters. Cluster 3 was characterized by higher expression of mitochondrial genes (*MTRNR2L12*, *MTRNR2L8*, *MT-ATP6, MT-ND4L*). This cluster most likely represented apoptotic or lysing cells, as cells with higher mitochondrial gene expression are more likely to die. Cluster 4 exhibited uniquely the expression of cell cycle genes (*ASPM, MKI67, GTSE1, NCAPG, CENPF, DLGAP5, HMMR*) indicating an association with cell division (Figure S3B and Table S2). Interestingly, an earlier study [27] also discovered a microglia cluster with proliferative signature using scRNA-Seq and human microglia purified from aged DLPFC implying this to be a naturally occurring state of microglia, rather than an artefact of our culture system.

### scRNA-seq reveals that iMG are more akin to human microglia than to human monocytes

To test the similarity of the iMG transcriptome to that of human primary microglia, we performed a comparative analysis.

Olah et al. [27] identified a set of genes that are specifically expressed in subsets of microglia freshly purified from aged postmortem human brains. Remarkably, all four of our iMG clusters expressed these microglial genes, including *C1Q, CABLES1, TREM2, ITGAM, PROS1, APOE, SLCO2B1, HAVCR2, TGFBR1, GPR34, RGS10, C3AR1, SLC7A8*, and *GAS6* (Fig. 3E) in contrast, we observed no expression of other glial genes (e.g., *GFAP, OLIG1, and OLIG2*) or neuronal (e.g., *RBFOX3, NEUN*, and *MAP2*) genes across any cluster (Fig. 3E right lower panels). Interestingly, our iMG expressed high levels of myeloid genes *SPI1* (PU.1), *RUNX1*, and *CSF1R* but showed very weak expression of monocyte-specific genes including *KLF2, VCAN, FCN1, TREM1, ITGAL, THBS1, IRF1, NR4A1*, and *KLF4* [27, 45–47] (Fig. 3E, right upper panel), suggesting iMG predominantly expressed microglial genes.

Next, we integrated iMG sc-RNA-seq data with the post-mortem single-nucleus RNA-seq (snRNA-seq) data [48] of aging frontal cortex from the Religious Orders Study and Rush Memory and Aging Project (ROSMAP) donors [30, 49, 50]. snRNA-Seq data from ROSMAP donors defined cellular types in aging human neocortex. Importantly, the transcriptomic signature of iMG aligned exclusively with neocortical microglia and not with other cell types such as astrocytes, endothelial cells, pericytes, vascular and leptomeningeal cells (VLMC), oligodendrocytes, neurons, and their subpopulations (Fig. 3F) in the human brain.

In addition, we performed principal component analysis (PCA) on microglial marker gene expression in our iMG, human adult [27] and fetal [28] primary microglia and various other iPSC-derived microglia from different differentiation methods [22, 51]. The transcriptomic profile of our iMG was similar to other iPSC-derived microglia, but it was remarkably akin to human primary microglia (Fig. 3G). Furthermore, a closer look at the whole transcriptome of iMG compared to adult human microglia [28] (Fig. 3H) and fetal human microglia [27] (Figure S3D) revealed a strong correlation between iMG and adult pMG (Kendall’s tau correlation coefficient (Tb) = 0.6826, p-value = 0.0), and iMG and fetal pMG (Tb = 0.6120, p-value = 0.0) transcriptomes, suggesting a significant similarity between iMG and pMG.

Importantly, initial scRNA-seq detected weak expression of microglial chemotactic receptors *CX3CR1* and *P2RY12* in 20-day differentiated iMG (Fig. 3E), yet, an additional four days in homeostatic media resulted in a robust induction of these genes (Fig. 3I). Of note, a previous scRNA-seq study [27] on human adult microglia also detected low mRNA levels of *P2RY12* (Figure S3C) in certain clusters.

#### Gene ontology and pathways analysis of scRNA-seq dataset reveals phenotypic heterogeneity within iMG

To gain further insight into the molecular pathways and biological processes associated with iMG, we used ShinyGO [52] to identify Gene Ontology (GO) terms enriched in the iMG cluster defining gene sets (i.e. top 25 genes upregulated in each respective cluster). GO terms associated with Cluster 1 were mainly associated with immune and inflammation (Fig. 3J and Table S3) while Cluster 2 enriched GO terms were related to migration and chemotaxis of immune cells (Fig. 3K, and Table S3). GO terms associated with Cluster 3 were related to mitochondrial functions (Figure S3E), while the Cluster 4 signature gene set showed enrichment in processes related to cell cycle and cell division (Figure S3F).

Taken together, scRNA-seq analysis of iMG revealed that many aspects of functional heterogeneity observed in human cortical microglia were recapitulated in our iMG, such as the transcriptional segregation of subsets enriched in migration, proliferation, and inflammatory response.

### Global proteome profiling of iMG confirms their similarity to pMG

**Fig. 4:**
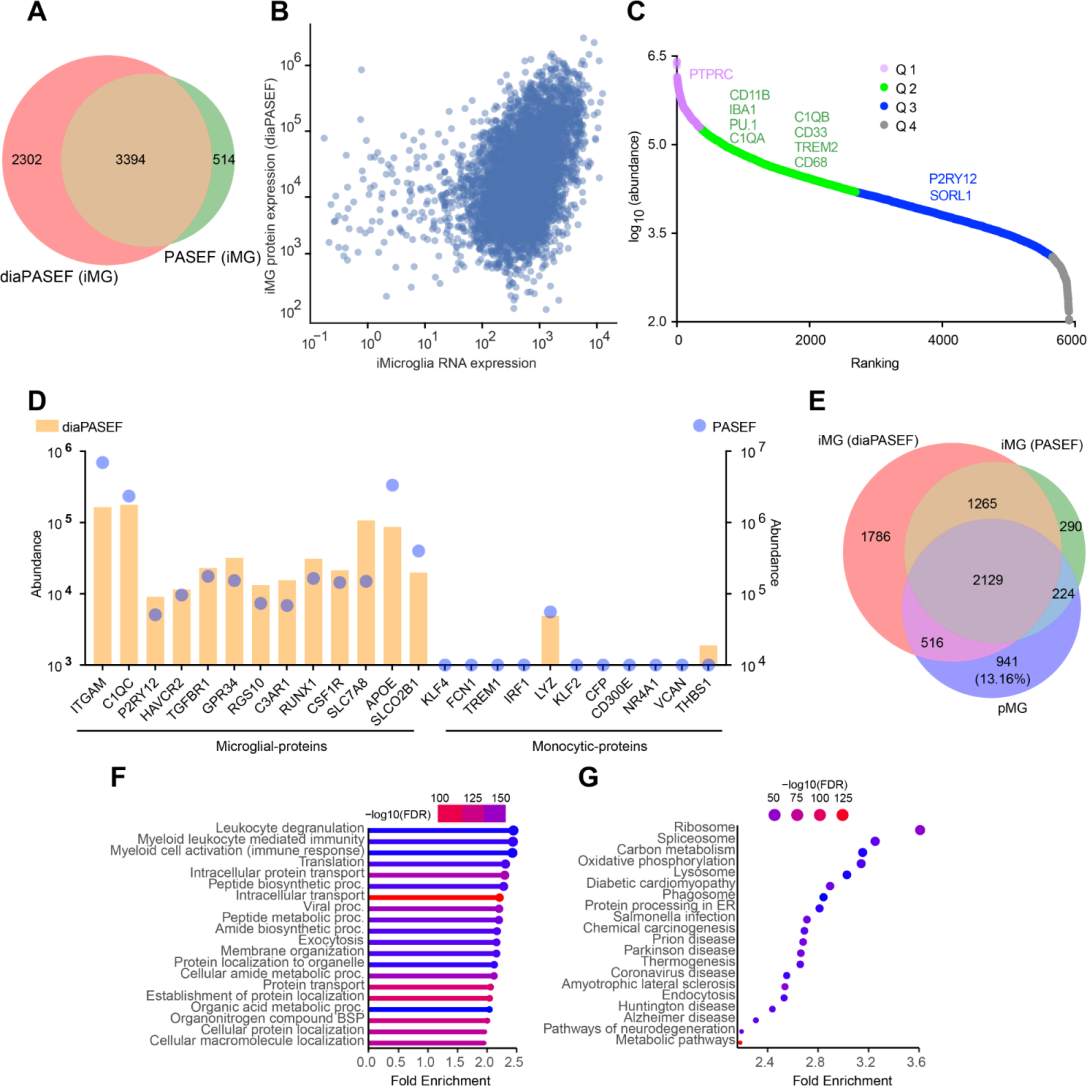
Global proteome profiling of iMG. **A**. Venn-diagram showing the number of proteins detected by diaPASEF and PASEF mass spectrometry (MS) in lysates from iMG (day 21). **B**. Dot plot showing the correlation between iMG mRNA expression (scRNA-Seq), and proteins expression measured in diaPASEF (T_b_= 0.2764, p-value = 5.550e^− 201^; Pearson’s *r* = 0.3794 and p-value = 6.1881e^− 182^). Each dot is a gene/protein pair. **C**. Scatter plot shows the abundance of proteins expressed in iMG. Global protein abundance was obtained using diaPASEF proteomics. On the graph, proteins are ordered based on their expression levels. Each dot represents a protein. The protein abundances were divided into four quartiles (Q: quartile). Microglial signature proteins were detected in quartile 1 and 2. **D**. Bar and dot hybrid plot showing the high expression of microglia signature proteins compared to the expression of proteins considered to be monocyte specific. Proteins abundance measured in diaPASEF methods is shown in the left y-axis while the right y-axis shows proteins abundance detected in conventional PASEF method. **E**. Venn-diagram is showing the comparison of iMG proteome with the proteome of 5000 pMG from a previous study (Olah, M. et al. 2018). Most of the proteins expressed in pMG overlapped with the proteome of iMG. **F**. GO analysis of most abundant proteins identified in iMG MS. Lollipop chart (ShinyGO) provides information about Biological Processes (BP), fold enrichment significance (FDR in log10) and number of genes (size of the circle) in each BP identified. **G**. Pathways analysis results of proteins identified in iMG MS. The most significant 20 pathways are shown. Dot plot shows the top 20 enriched pathways, significance (FDR in log10), and the number of genes in each pathway shown. Cellular lysate from C1-iPSC line was used for MS. Abbreviations: PASEF parallel accumulation serial fragmentation; diaPASEF data independent acquisition parallel accumulation serial fragmentation; iMG induced pluripotent stem cell derived microglia; pMG primary microglia; FDR false discovery rate

In addition to the transcriptome analysis, we assessed for the first time the global proteome of iMG produced by the two-step protocol, using mass spectrometry (MS). We collected 0.4 million iMG on day 21 (Fig. 1A) for MS sample preparation. We used two acquisition methods (parallel accumulation-serial fragmentation (PASEF) [31] and parallel accumulation-serial fragmentation combined with data-independent acquisition (diaPASEF) [32] for bottom-up/shotgun proteomic analysis of iMG. Conventional PASEF is dependent on ion threshold where higher abundant ions get fragmented in the cycle time, while diaPASEF exhibits fragmentation of every ion in the specified window size, hence giving deeper proteomics coverage and data consistency. Indeed, compared to PASEF, diaPASEF detected a higher number of iMG proteins with at least one peptide (5,696 vs. 3908), although most microglial proteins are detected by both methods (Fig. 4A).

We also observed a moderate correlation between mRNA levels and proteins detected in diaPASEF (Fig. 4B) (Tb = 0.2764, p-value = 5.550e-201. Pearson’s *r* = 0.3794 and p-value = 6.1881e-182) and PASEF (Figure S4A) acquisitions. The mRNA levels were estimated by pooling read counts in all cells in the scRNA-seq data.

Next, based on the relative abundance of detected proteins, we divided the MS-identified proteins into four quartiles. Microglia signature proteins including PTPRC, FTL (ferritin), CD11B, IBA1, PU.1, C1QA, C1QB, CD33, TREM2, PTGS1 (COX1), CSF1R and CD68 were detected in quartile 1 and 2 (most abundant), while homeostatic microglial marker P2RY12, which senses extracellular nucleotides from degenerating neurons [53], was detected in quartile 3 (Fig. 4C and Figure S4B). MHC II proteins including HLA-DRA, HLADRB1 were detected in quartile 2. iMG expressed high levels of microglial surface receptors (e.g., CD14, CD40, CD74, and CD86), as well as phagocytic receptors including FCGR1A, FCGR2A, FCGR3A, CD68, CD36, CD163, CD33 CD45 and TLR2 (quartile 1 and 2) (Figure S4C).

Interestingly, while scRNA-Seq detected weak expression of CX3CR1 and P2RY12 transcripts without using homeostatic media (Fig. 3E), MS reliably detected P2RY12 protein (Fig. 4C) in iMG at day 21 of differentiation. While the difference may be explained by sensitivity and technical details of the two assays, it is also possible that there is extensive regulation of translation efficiency and protein stability in human iMG, as suggested for human pMGs [54].

It has been shown that in some iMG differentiation protocols hiPSCs may differentiate into monocytes [16]. We compared the expression of microglia-enriched proteins to proteins known to be enriched in monocytes. Remarkably, our iMG predominantly expressed microglia-enriched proteins but not monocyte-specific proteins (Fig. 4D). Consistent with ATAC-Seq and scRNA-Seq data, MS did not detect any peptide of pluripotent markers (e.g., POU5F1, OCT4 and NANOG) in iMG, suggesting a complete microglial differentiation of hiPSCs.

A previous study [24] identified the proteome of 5000 primary microglia purified from aged human dorsolateral prefrontal cortex (DLPFC). We compared the proteome of our iMG with proteome of aged primary human microglia (pMG) described in previous study [24]. Although smaller number of FACS sorted pMG (5000) were measured in the previous study, the proteins identified in our iMG MS covered most of the proteome (> 86%) of primary human microglia (Fig. 4E). pMG versus diaPASEF, T_b_= 0.2804, p-value = 4.3608e^− 80^. Pearson’s *r* = 0.2146 and p-value = 6.8229e^− 23^, pMG versus PASEF, T_b_= 0.2704, p-value = 1.0337e^− 69^. Pearson’s *r* = 0.2658 and p-value = 4.8032e^− 32^.

The gene ontology and pathways enrichment analysis of the detected proteins identified GO terms including leukocyte degranulation, myeloid leukocyte mediated immunity and myeloid cell activation (immune response) while enriched pathways included phagosome and neurodegeneration related pathways (Fig. 4F-G). The list of proteins detected in PASEF and diaPASEF proteomics are provided in supplementary data as Tables S4 and S5, respectively.

Collectively, the global proteome analysis suggests that iMG bear a significant resemblance to primary human microglia.

### iMG phagocytose substrates relevant to neurodegeneration

**Fig. 5:**
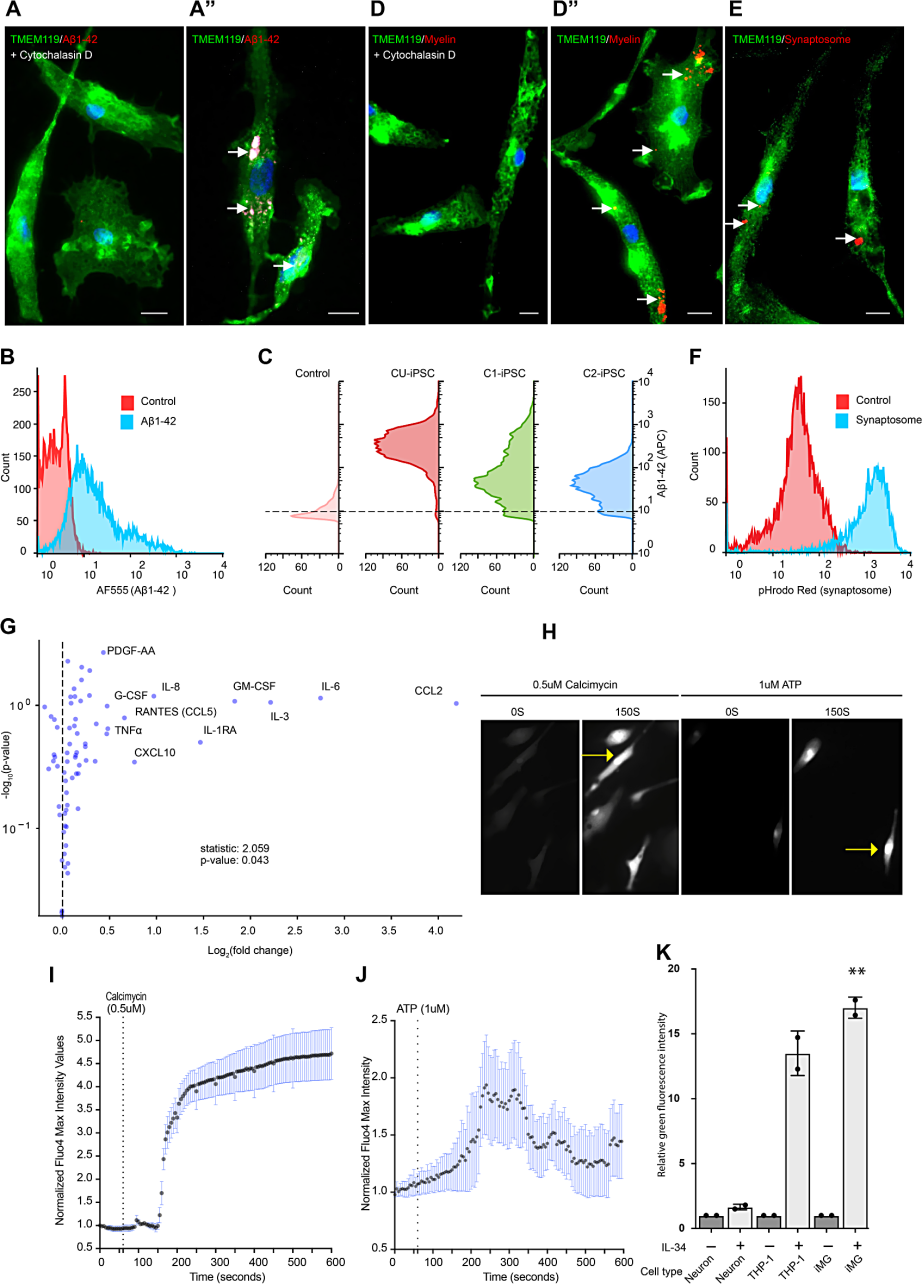
Functional validation of iMG microglial identity. **A**-**F**. Phagocytic activity in iMG. **A**-**A”.** Microscopic images showing iMG (C1-iPSC) phagocytosing Flour 555-labeled beta-amyloid (**A”)**, which is inhibited by cytochalasin D (**A**). **B**. Flow cytometric analysis of Flour 555-labeled beta-amyloid treated iMG derived from C1-iPSC line. Red histogram shows the control sample (untreated), and light blue histogram shows the sample exposed to Flour 555-labeled beta-amyloid. **C**. Flow cytometry of fluorescent beta-amyloid treated iMG differentiated from three different iPSC lines. Control: cytochalasin D treatment. (**D**-**D”**) iMG phagocytosing pHrodo-red labeled myelin with (**D**) and without cytochalasin D (**D”**). **E**-**F.** iMG engulfing pHrodo-red labeled human synaptosomes, confirmed by microscopy (**E**) and flow cytometry (**F**). Red histogram shows the control sample and light blue histogram shows the sample exposed to pHrodo labeled synaptosomes but not cytochalasin D. **A**-**F** n = 3. A, A’’, D, D’’, E: CU-iPSC. C: all three iPSC lines. Scale bars: 10 μm in A-A”, D-D” and E. **G**. iMG response to LPS challenge by releasing cytokines and chemokines. The volcano plot shows the means of three independent experiments of LPS or vehicle-only treatment for 12 h. A two-sided paired t-test for each cytokine/chemokine was applied to assess if LPS treatment had an overall impact on cytokine/chemokine secretion (*P* < 0.05). **H**. A23187 and ATP evoke Ca^2+^ transients in iMG, as depicted by live imaging of Fluo-4 labeled iMG following addition of either compound. Arrows highlight Ca^2+^driven signal accumulation in iMG over time. **I**-**J**. Image-derived fluorescence intensity signal measurements over time post A23187 (**I**) or ATP (**J**) treatment. Recordings began 60 s after baseline measurements and continued for 600 s. Supplementary **Figure S5D** shows the recording up to 1800 s. Error bars in I and J express the standard error of means of three independent experiments. The full registry of Ca^2+^ dynamics in video format can be found in the supplementary material. **K.** iMG migrate toward IL-34 cytokine, as demonstrated by fluorometric InnoCyte™ cell migration assay results, represented as relative green, fluorescent intensity (GFI). Neuron: SH-SY5Y neuronal cell line. Error bars represent the standard deviation of three independent experiments (iMG vehicle-only vs. iMG IL-34; Student’s t-test *p* = 0.0013). Abbreviations: Aß amyloid beta; AF555 AlexaFluor555, a fluorochrome; APC allophycocyanine, a fluorochrome; THP-1 a monocytic cell line; iMG induce pluripotent stem cell derived microglia

Microglia contribute significantly to amyloid beta (Aβ) clearance in the aged brain [55]. Notably, Aβ deposition, especially that of Aβ_1−42,_ characterizes AD [56, 57]. We evaluated the capability of our iMG to phagocytose Aβ_1−42_ by incubating them with fluorescently labeled Aβ_1−42_. Both microscopic examination (Fig. 5A-A”) and flow cytometry analysis demonstrated that, iMG differentiated from all three iPSC lines utilized in this study, could internalize fluorescently labeled Aβ_1−42_ (Fig. 5B-C). Additionally, the engulfment of this fluorescently labeled Aβ_1−42_ by iMG was further verified through a 3D analysis using Z-stacks of confocal images (see extended data Figure Ext. 2). Importantly, the treatment with cytochalasin D, a potent phagocytosis inhibitor that interferes with actin polymerization [58], nearly eliminated the phagocytosis of Aβ_1−42_ (Fig. 5A C), confirming the validity of our phagocytosis assay. This implies the potential of these iMG to be used in high throughput screening tests to identify therapeutic drugs and genes that can influence the clearance of Aβ_1−42_.

Microglial phagocytosis also plays an essential role in the clearance of myelin debris in the brain. We further tested the functionality of our iMG by exposing them to pHrodo Red-labeled myelin purified from post-mortem human DLPFC. Of note, pHrodo Red is a pH-sensitive dye that emits bright red light in the low pH of post-phagocytic phagolysosome compartments. The lack of fluorescence outside the cell thus enables robust quantification of cellular uptake with a clean background [59]. Incubating iMG with pHrodo Red-labeled myelin revealed robust pHrodo Red-positive phagosomes (Fig. 5D-D” and Figure S5A). However, cytochalasin D treatment inhibited phagocytosis of pHrodo Red-labeled myelin (Fig. 5D), confirming iMG’s capability to phagocytose human myelin.

Another critical role of microglia in health and disease is synaptic pruning [60, 61] in the brain. To test whether iMG phagocytose human synaptosomes, we prepared synaptosome enriched fraction (Figure S5B) from DLPFC of human post-mortem brain, using a previously described protocol [34]. Following extraction, we labeled synaptosomes with pHrodo Red. Incubation of iMG with pHrodo Red-labeled synaptosomes resulted in efficient internalization of synaptosomes as shown by both microscopic observation (Fig. 5E) and flowcytometric analysis (Fig. 5F).

In conclusion, iMG demonstrated efficient uptake of diverse substrates found in the human brain, confirming that iMG display essential functions of primary microglia.

#### iMG produced cytokines in response to inflammatory signal

Microglia, the primary mediators of neuroinflammatory processes in the CNS, respond to various pathological changes, including injury, ischemia, and infection, by expressing a range of cytokines like IL-1β, IL-6 and TNFα.

We investigated the cytokine profiles released by iMG after stimulation with lipopolysaccharide (LPS) or vehicle control. Utilizing a multiplex array to measure cytokine concentrations in the supernatants, we observed that post LPS treatment, iMG exhibited increased release of several proinflammatory (IL-6, IL-1β, TNFα, TNFβ, IFNγ, IL-33, IL-12p70, IL-1RA, TPO, IL-17F, and IL-17A) and anti-inflammatory (IL-10 and IL-27) cytokines (Fig. 5G, Table S6). The expression levels of several cytokines involved in the adaptive immune response, including IL-5, M-CSF, IL-4, IL-9, IL-21, and IL-15 were also elevated.

Furthermore LPS-stimulated iMG released several chemokines (CCL5, CCL7, CCL3, CXCL10, CCL8, CXCL1, MIG/CXCL9, CXCL5 (ENA-78), CCL1(I-309), CCL17 (TARC) and CXCL8 (IL-8) responsible for regulating inflammatory cell influx into the brain (Fig. 5G). Interestingly, LPS-treated iMG retracted their processes and changed morphology to rounded (Figure S5C), suggesting the recapitulation of a primary microglial feature [62].

These data imply that iMG generated through our protocol display a robust inflammatory response to pathogenic substances, such as LPS, a key feature of human primary microglia.

#### Nucleotide stimuli evoke Ca^2+^ transients in iMG

In response to neuronal damage or degeneration in the brain, microglia become active, morphing into an ameboid form that can migrate to the damaged neurons [63]. Multiple studies [64–66] have demonstrated that adenosine 5′-triphospshate (ATP) and other nucleotides are released into extracellular space because of injury-related cell death. Once in the extracellular space, the nucleotides behave as “danger signal” and can directly activate membrane bound P2X and P2Y nucleotide receptors on microglia [67]. The activation of these receptors initiates intracellular signaling cascades through Ca^2+^ activity, that regulates microglial functions. A well-documented response to ATP in primary microglia is the immediate Ca^2+^ transient which guides microglia migration to the injury site [67–69].

We evaluated the intracellular Ca^2+^ activity of our iMG in response to ATP and calcimycin (A23187) an ionophore widely used to study intracellular Ca^2+^ dynamics. We used green fluorescent (excitation/emission 494/506 nm) calcium indicator Fluo-4 for the detection of Ca^2+^ flux by imaging. Fluo-4 is an analogue of Fluo-3, which is an essentially nonfluorescent compound, but upon binding of Ca^2+^ its fluorescence increases sharply [70]. Quantitative image-based fluorescence registries (Fig. 5H) showed a strong and reproducible Ca^2+^ transient activity in iMG, after stimulation with A23187 (Fig. 5I) or ATP (Fig. 5J). Both stimulations increased the Fluo-4-driven signal rapidly (Supplementary video V1 and Supplementary video V2, respectively), followed by a plateau phase. However, A23187 induced a higher Fluo-4 fluorescence amplitude and longer plateau phase compared to ATP (Figure S5D). This evidence suggests that iMG exhibit ATP-evoked Ca^2+^ transients, a key characteristic of primary microglia.

For the ATP stimulation experiment (1µM), we captured images at intervals of 0, 150, 300, 450, and 600 s. In the case of 0.5mM calcimycin, the time points were 0, 300, 600, 900, and 1145 s. These images chronicle the progression from stimulation to signal decay, with representative images of Ca2 + recovery featured in extended data (Figure Ext. 3). Furthermore, the extended data encompasses a comprehensive graph of the calcimycin experiment, tracking calcium activity from 0 to 1145 s, including the phase of signal decay (Figure Ext. 4) .

#### iMG exhibit directional migration toward chemotactic signals

Primary microglia are highly mobile, continuously migrating throughout CNS development and in response to damage-associated signals. The motility of microglia is regulated by chemoattractants and various signaling cascades including receptors and kinases. To investigate if iMG sense and migrate towards a chemotactic cytokine gradient, we performed InnoCyte™ assay using IL-34 as the chemotactic agent. IL-34 can trigger macrophage [71] and primary microglial [72] migration through Syndecan-1 and CSF1RA pathways, respectively. Given our iMG showed high expression level of CSF1R (Figs. 5E and 4D), we utilized the CSF1R ligand IL-34 to assess these cell’s migratory and invasive capacities. CSF1R/IL-34-based InnoCyte™ cell migration assays showed that the number of iMG migrating across the chamber was significantly higher in the presence of 20 ng/mL IL-34 in the attractant chamber, while control cells (SH-SY5Y neuronal cell line) showed no significant difference (Fig. 5K). These results indicate that our iMG display directional migration towards a gradient of a cytokine that is known to be a chemoattractant for human microglia, further corroborating their similarity to primary microglia.

#### CRISPR/Cas-mediated gene knockdown and overexpression in iMG

Genetic studies of numerous neurodegenerative diseases [73–76] including AD have identified microglia as crucial player in disease development and progression. Yet, what is the role of these implicated microglia genes in microglia biology and how they contribute to the pathogenesis and pathobiology of neurodegenerative diseases, remains unclear. While CRISPR/Cas systems offer tools to decipher the function of disease-associated genes, fundamental challenges such as lengthy microglial differentiation protocols, and difficulties to efficiently insert CRISPR/Cas vectors or synthetic proteins into mature microglia, impede progress in the field.

To address these challenges, we developed a tetracycline-regulatable CRISPR interference (Tet-CRISPRi) and CRISPR activation (Tet-CRISPRa) systems in our iMG to enable robust knockdown and overexpression of endogenous genes. Tet-CRISPRi and CRISPRa systems offer gene expression manipulation with temporal control.

**Fig. 6:**
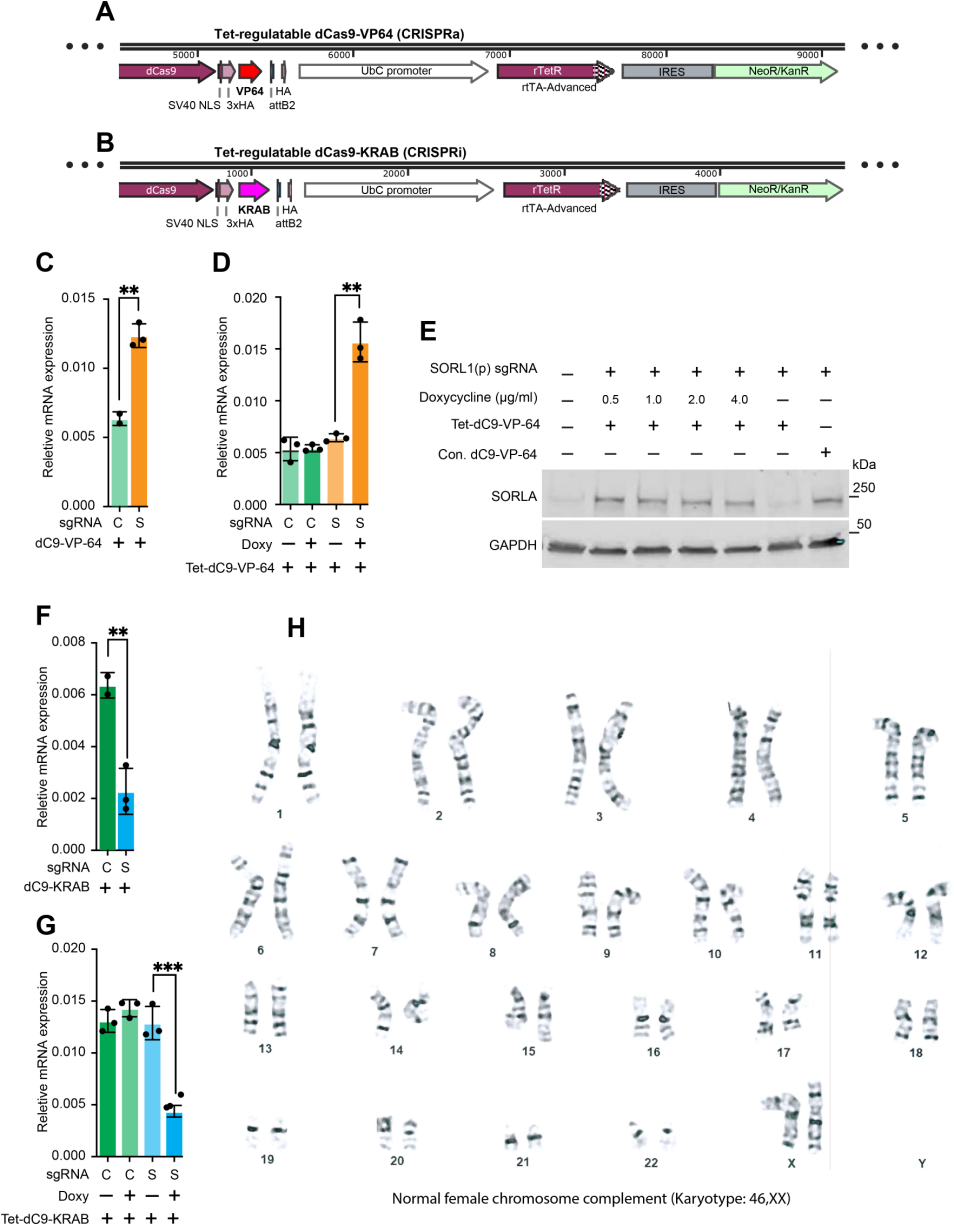
CRISPR-dCas9 mediated gene activation/repression in iMG. **A** & **B**. Schematic for drug-inducible (tetracycline/doxycycline) dCas9-VP64 (activation) and dCas9-KRAB (repressor) expression vectors, respectively. Target sgRNAs were expressed with Tet-dCas9-VP64 or Tet-dCas9-KRAB domains at iPSCs stages, while doxycycline was added at day 16 of differentiation. **C**. Quantitative analysis of mRNA expression of *SORL1* by RT-qPCR, after lentiviral expression of control (C) and *SORL1* promoter (S) targeting sgRNAs in iPSCs with stably integrated constitutive dCas9-VP64 (mean ± SD, *n* = 3, t test *P* = 0.0032). **D**. Functional validation of doxycycline-inducible dCas9-VP64 (CRISPRa) via quantitative PCR on *SORL1* mRNA levels in iMG expressing *SORL1* promoter targeting sgRNA or control sgRNA (mean ± SD, *n* = 3, ANOVA, *P* < 0.0001). Doxycycline was added on day 16 of differentiation. **E**. Immunoblotting demonstrates overexpression of SOLR1 depends on doxycycline treatment after lentiviral deliveries of Tet-dCas9-VP64 and sgRNA targeting *SORL1* promoter (*SORL1*(p)). (Con. dC9-VP-64 stands for: constitutive expression of dCas9-VP64). The blot is representative of three independent experiments. Complete western blot gel is provided in supplementary Figure S7. **F**. RT-qPCR analysis of mRNA expression of *SORL1*, after lentiviral expression of control (C) and *SORL1* promoter (S) targeting sgRNAs in iPSCs with stably integrated constitutive dCas9-KRAB domain (*P* < 0.05, (mean ± SD, *n* = 3, t test *P* = 0.01). **G**. Drug-inducible dCas9-KRAB (CRISPRi) represses *SORL1* transcription only when doxycycline is added to the cell culture. The bar chart shows the quantitative difference in mRNA levels, measured by RT-qPCR, in iMG expressing *SORL1* promoter targeting sgRNA or control sgRNA (mean ± SD, *n* = 3, ANOVA, *P* < 0.0001). Doxycycline was added on day 16 of differentiation. **H**. G-banded chromosome analysis shows a normal karyotype of differentiated iMG

Taking advantage of the currently available drug inducible vectors [38, 77, 78] we stably introduced into hiPSCs cultured in parallel dishes the doxycycline inducible catalytically inactive Cas9 (dCas9) coupled with KRAB repressive domain (Tet-CRISPRi) and dCas9 coupled with VP64 activation domain (Tet-CRISPRa). Additionally, we integrated constitutive CRISPRi/a machinery into hiPSCs to compare with inducible CRISPRi/a efficiency. Following lentiviral infections of hiPSCs, we confirmed constitutive or doxycycline-dependent expression of CRISPRi/a under control of EF1-alpha or tetracycline-responsive (TRE) promoters respectively (Fig. 6A-B and Figure S6A-B).

To evaluate the efficiency of both constitutive and tet-regulatable CRISPRi/a in iMG, we aimed to manipulate the expression of AD linked gene *SORL1* in these cells. The protein encoded by *SORL1*, known as SORL1 is a type-I transmembrane protein associated with the regulation of intracellular transport and processing of Aβ precursor protein in neurons. Its role in microglia, however, remains largely unknown.

We used FANTOM [79] data to identify *SORL1’*s transcription start site (TSS) and designed single guide RNAs (sgRNA) for CRISPRi and CRISPRa (Figure S6C).

For CRISPRa, we delivered *SORL1* sgRNAs or a control sgRNA to the dCas9-VP64 and tet-dCas9-VP64 expressing hiPSCs, using a modified lentiviral plasmid [36] carrying a gRNA scaffold, a U6 promoter and the transcriptional activation domain P65-HSF1. Three days post sgRNA expression, iMG expressing constitutive CRISPRa showed more than 194% increase in *SORL1* expression (Fig. 6C) while those expressing inducible CRISPRa exhibited more than 280% increase in *SORL1* transcription, but only after doxycycline treatment (Fig. 6D). In both systems the increase in *SORL1* transcription was sufficient to allow the accumulation of SORL1 protein detected by western blot analysis (Fig. 6E and Figure S6D).

Similarly, we examined the efficiency of the inducible CRISPRi system by studying the repression of the endogenous *SORL1* expression (Fig. ;6B). hiPSCs with constitutive dCas9-KRAB expression showed a robust repression (> 357%) of *SORL1* transcription following sgRNA expression (Fig. 6F and Figure S6E). In the cells with Tet-dCas9-KRAB, *SORL1* repression (> 320%) was dependent on sgRNA expression and doxycycline treatment (Fig. 6G). These results suggest that our inducible CRISPRi/a systems are efficient and can be utilized to study gene function in our iMG model system.

#### Two-step iMG differentiation protocol or CRISPR-ON/OFF system does not affect genomic integrity of iMG

Chromosomal abnormalities can arise in hiPSCs during cell culture, differentiation, or genome editing [80, 81]. For instance, gains of chromosome 12, 17, 20 or X have been reported in iPSCs following long-term culturing [82]. Although biological significance of these chromosomal abnormalities remain under debate, they may confer a strong selective growth advantage to the cultured cells [83]. Therefore, ensuring a normal karyotype is a key requirement for generation of iMG from hiPSCs. Previous iMG differentiation protocols are lengthy and may affect genomic integrity of resulting iMG. Similarly, conventional CRISPR-Cas9 systems to edit genes in living cells introduce a double-stranded DNA break, which can induce complex genomic rearrangements in target cells [84].

Although our protocol requires only 20 days to generate iMG, and we employed next generation CRISPR-dCas9 system that circumvents DNA double-strand breaks, we sought to determine whether our differentiation protocol or gene ON/OFF CRISPR-dCas9 system impacts the karyotype of resulting cells. We conducted a karyotype test on iMG chromosomes using cytogenetic G-banding approach. As anticipated, neither the inducible ON/OFF CRISPR-dCas9 system nor our 20-day iMG differentiation protocol affected the genomic integrity of the cells (Fig. 6H). The analysis of the banding pattern did not detect any chromosomal abnormalities such as polyploidies, aneuploidies, or any large balanced alterations in iMG chromosomes, suggesting a normal karyotype of the cells.

#### Searchable, web-accessible platform for exploring our iMG model system’s multi-omic dataset

To provide an in-depth characterization of our iMG model system, we carried out a comprehensive multi-omic analysis, incorporating scRNA-Seq, ATAC-Seq and proteomics on our iMG. We also conducted ATAC-Seq on primary human microglia sourced from DLPFC for comparison. We compiled these data sets into a web-based, user-friendly, freely searchable platform for the scientific community to easily access and evaluate this iMG model system.

Our online platform provides a graphical comparison of iMG with pMG, including data from ATAC-Seq and proteomics. The platform is divided into four primary sections: gene expression, ATAC-seq browser, proteomics, and cytokines release.

**Fig. 7:**
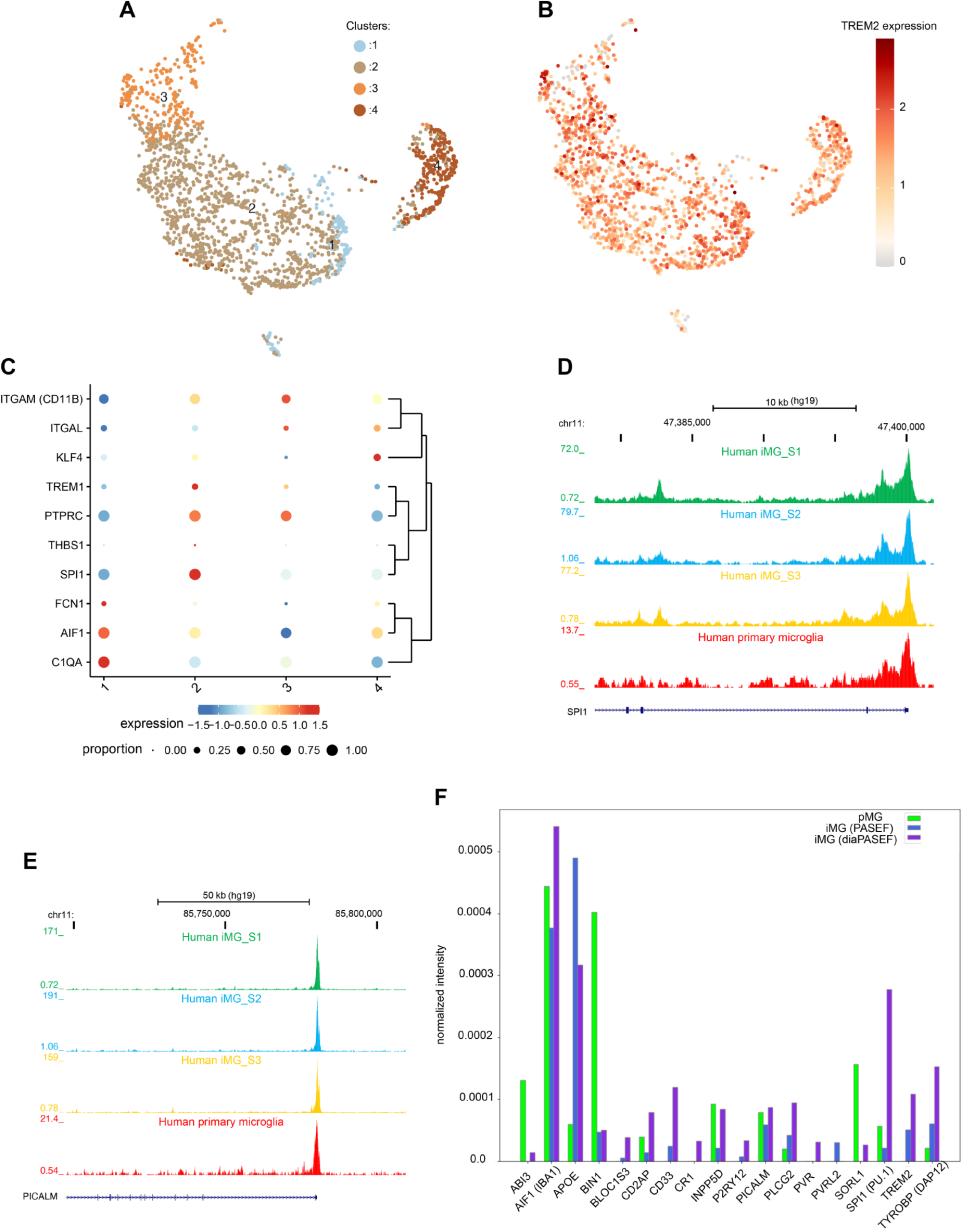
A web-accessible platform showcasing graphical visualization of the multi-omic data from iMG model system. **A**. Depicts how the web portal displays the K-means clustering of ~ 2000 single-cell transcriptional profiles from iMG. **B**. Displays, as an example the expression of TREM2 gene in all four identified K-means clusters. **C**. Web-platform offers user-defined visualization of average, log-transformed expression of multiple genes. Depicted are 10 microglial gens, grouped by categorical cell information (i.e. cluster identity). **D**-**E**. Show ATAC-Seq data browsing in the UCSC genome browser, demonstrating density plots around AD-linked genes SPI1 (**D**) and PICALM (**E**) in iMG (top three tracks) and pMG (bottom track). **F**. Demonstrates comparative abundance of microglial proteins in iMG and pMG using mass spectrometry data, displayed after normalizing individual protein abundances by the sum of all abundances

In the gene expression section, users can examine gene expression profiles either by cell cluster or by gene search (Fig. 7A-B). Various formats such as violin plot/boxplot, proportion plot or bubble plot/heatmap are also available for viewing gene expression data. The bubble plot/heatmap function (Fig. 7C) allows user to view the expression patterns of multiple genes, organized by categorical cell information (microglia cell clusters). All expressions are depicted as averaged (normalized), log-transformed. Importantly, user can control cell subsets, and graphical output.

ATAC-Seq section offers user the opportunity to examine the ATAC-Seq data of iMG and pMG directly on UCSC genome browser, for an in-depth examination of regions of interest (Fig. 7D-E).

The proteomic plot section provides access to proteomic data of iMG (PASEF and diaPASEF) and pMG, enabling user to compare the normalized intensity of individual or grouped proteins expressed by iMG and pMG (Fig. 7F).

Cytokine plot section displays the fold change in cytokines/chemokines released in the supernatant of LPS-stimulated iMG.

Our intuitive, web-accessible platform aims to expedite research by providing a searchable phenotypic database for our iMG model system. This platform and the associated optimized protocols will enable researchers to set up well-informed functional genomic experiments.

## Discussion

Several existing protocols generate microglia from human induced pluripotent stem cells (hiPSCs), but most of them are associated with challenges such as a lengthy differentiation processes, that can have undesirable effects on the resulting cell’s genomic integrity. They also often present an incomplete characterization of produced iMG. Comprehensive proteome, DNA accessibility sites, and other relevant profiles are usually missing, while transcriptomic data sets that are provided require bioinformatics skill to analyze. Though some shorter protocols exist, they use plasmids and antibiotics which can negatively impact homeostatic properties of the resulting iMG.

**Fig. 8:**
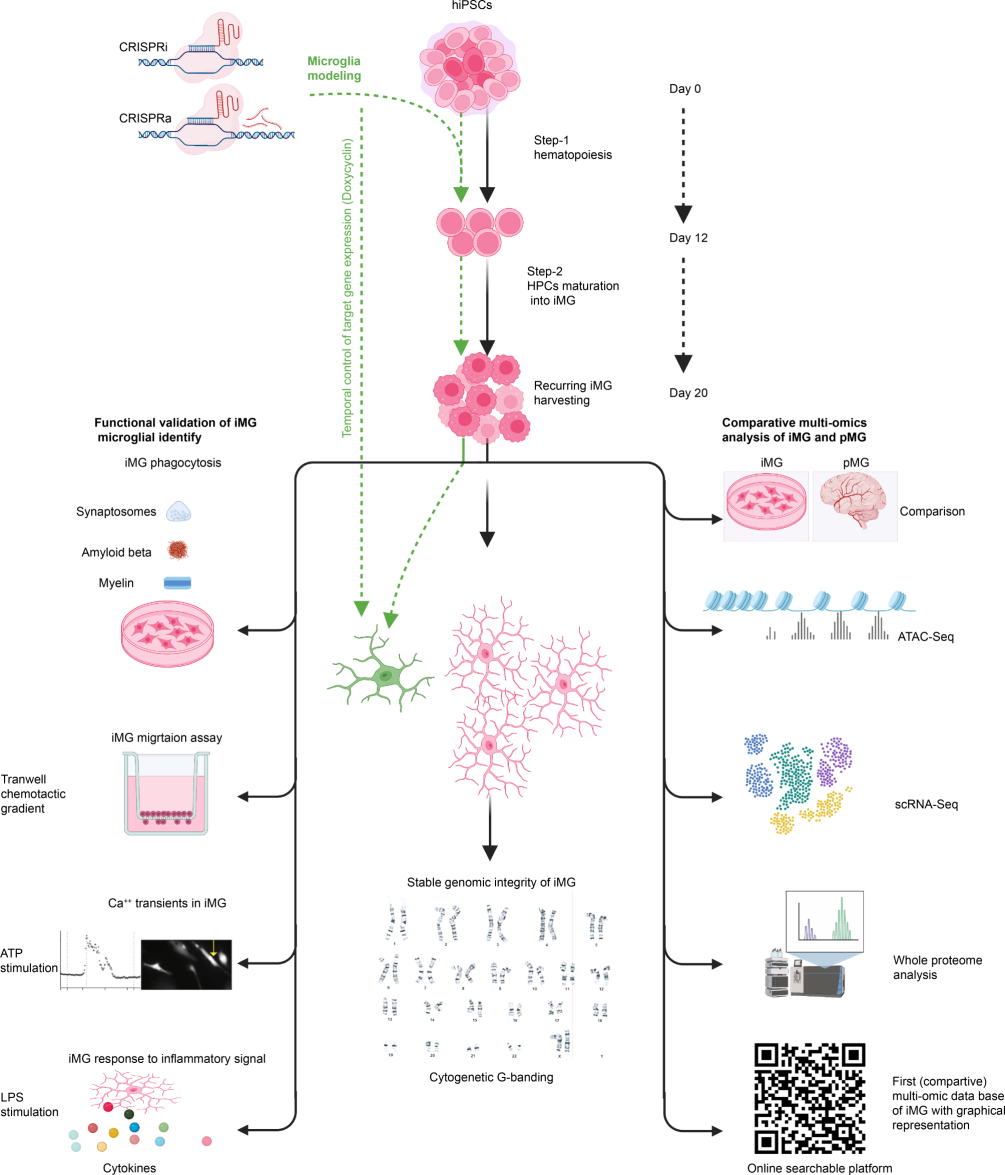
Integrated toolkit for functional genomics of human microglia. Schematic illustrates the process of iMG generation, manipulation of gene expression, and comprehensive characterization of iMG. The graphics in this Figure were generated using BioRender

Thus, a need for a deep phenotyped iMG resource exists in the field. We sought to address this by providing a comprehensive, user-friendly online database of iMG transcriptomic, proteomics, DNA accessibility, and cytokine secretion profiles of iMG to the scientific community. Moreover, we wanted to streamline the differentiation process to maintain genomic integrity and allow continuous progenitor proliferation for recurring collection of freshly formed iMG. In response to these needs, we have developed an integrated functional genomics toolkit for microglia (Fig. 8). Our toolkit offers an efficient two-step method to produce pure human microglia-like cells (iMG) from hiPSCs, with stable karyotype within 20 days. It includes in depth validation through single-cell RNA-Seq, ATAC-Seq, proteomics, comparisons with primary human microglia, a standardized set of assays for functional assessment. Furthermore, it features a stand-alone user friendly, web-accessible platform, hosting graphical representations of our iMG model system’s data. In addition to the step-by-step protocol for iMG differentiation from hiPSCs, an optimized protocol for the deployment of a drug-inducible CRISPR-dCas9 system in iMG is also included. This system allows temporally controlled gene function investigation and will be useful for the research groups who have less experience in iPSCs and gene editing technologies. Importantly, our iMG differentiation protocol utilized a recurring iMG harvesting approach, allowing the large-scale production of iMG for high-throughput assays.

The iMG produced using our protocol resemble primary human microglia more closely than monocytes, based on transcriptomic, DNA accessibility and proteomics analyses. They also demonstrate characteristic microglial functions, such as inflammatory response to LPS, phagocytosis, Ca2 + transients in response to nucleotide stimuli, and migration. Notably, our CRISPR-ON/OFF system efficiently manipulate gene expression without causing karyotypic changes.

Microglia play critical roles in brain development and function, with their altered functions linked to various brain pathologies. Studying microglia purified from human brain is challenging due to the scarcity of material and our inability to control for comorbidities in ex vivo studies [24, 85–88]. The limited yield of viable cells post-isolation and changes in microglia behavior in culture also limit in vitro experimentation using human brain origin microglia [89, 90]. Some researchers derive microglia like cells from human monocytes [91–93]. Nonetheless, this approach remains divisive due to developmental differences between the origin of microglia and monocytes [85]. Mouse models, although useful for certain functional studies, are not particularly suited for neurodegeneration related functional genomic studies due to the differences in gene expression [94] (e.g. lacking certain AD-associated genes) and aging between human and mouse microglia [95]. The complement system (CR), important in neurodegenerative diseases, also varies between species [96]. Consequently, developing a reliable in vitro human microglia model with deep characterization is crucial for exploring both healthy and disease-related microglial phenotypes and pathways.

Recent efforts to convert hiPSCs into iMG have utilized overexpression of key microglial transcription factors. Chen et al. induced hiPSCs into iMG in < 20 days with SPI1/CEPBA overexpression. However, only a small fraction (4.5-25%) of input hiPSCs followed differentiation into iMG [15]. A very recent protocol [22] induced iMG differentiation by integrating six transcription factors into hiPSC using CRISPR-Cas9. Although the approach is very innovative, the use of multiple vectors and drugs for selection of populations of interest limit the downstream utility of produced iMG.

Several research groups have made strides in developing microglia from human iPSCs, mimicking embryonic development, but these methods significantly vary in terms of starting cell type, culturing conditions, differentiation time, purity, and properties of the resulting microglia. Muffat and colleagues [12] generated iMG from hematopoietic cells derived from embryoid bodies (EB). Although the resulting mature iMG from this protocol showed resemblance to freshly isolated fetal human microglia, the total culture time was 75 days, and resulted in impure microglia due to EB contamination. Pandya et al. [13] used FACS to enrich for CD34^+^/CD43^+^ cells and obtained iMG yet faced issues with scalability and purity. Other protocols [11, 17, 18, 20, 97] also differentiated hiPSCs into microglia-like cell, but they are lengthy (> 40days) and variable in yield and gene expression [16, 22]. Building on previous protocols e.g. Muffat et al. [12] and Pandya et al. [13] more recent approaches [14] obtained iMG by co-culturing differentiating hematopoietic progenitors and astrocytes, however resulting iMG carried astrocytic impurity originating from the co-culture system. Furthermore, these approaches partially fulfill the requirements for certain in vitro quantitative assays that need large number of relatively pure iMG.

We improve on these methods by utilizing commercially available kits and human astrocyte conditioned medium to increase reproducibility, purity, and yield of the resulting iMG, while significantly reducing differentiation time. Our toolkit also includes a standalone searchable online database with graphical representation of our iMG model system data, and a detailed technical description of the differentiation process and drug-inducible CRISPR-ON/OFF systems.

The choice to conduct scRNA-Seq and proteomics on day 20 iMG was driven by technical reasons. At this stage, iMG are floating and express key microglia genes, suitable for single-cell RNA-Seq and proteomics procedures. While subculturing iMG on adherent surfaces for three days in homeostatic media can enhance CX3CR1 and P2RY12 expression, trypsinization for subsequent scRNA-Seq/proteomics can introduce technical hurdles. This additional step risks clumping, increased cell death, and higher doublet ratios in scRNA-Seq data. Therefore, to ensure streamlined and reliable single-cell analysis, day 20 floating iMG were chosen. However, for experiments requiring homeostatic conditions and morphological assessment, adherent subcultures were established in homeostatic media. This approach effectively balanced the need for technical accuracy with the ability to study iMG function and morphology.

## Conclusions

Our integrated functional genomics toolkit offers a comprehensive approach to studying human microglia, effectively addressing several challenges of existing methods. Our advanced two-step method efficiently produces pure functional iMG from human iPSCs in 20 days, without compromising the karyotype of the resultant cells. The optimized CRISPR-ON/OFF system modulates gene expression with temporal control, and importantly, without inducing DNA double strand breaks. By utilizing commercially available resources and astrocyte conditioned medium, our method ensures enhanced yield and reproducibility. The toolkit provides robust multi-omic dataset, standardized functional assessment assays, and an easily navigable web platform. A significant feature is our recurring cell harvest approach, rendering this advanced iMG generation method apt for assays necessitating large cell quantities, such as high-content CRISPR-Cas9 screens. Furthermore, by enabling detailed AD-associated single nucleotide variants (SNV) investigation, our toolkit holds the potential to markedly propel neurodegenerative diseases research forward and aid in the development of therapeutics targeting neuroinflammation.

### Electronic supplementary material

Below is the link to the electronic supplementary material.

Supplementary Material 1

Supplementary Material 2

Supplementary Material 3

Supplementary Material 4

Supplementary Material 5

Supplementary Material 6

Supplementary Material 7

Supplementary Material 8

Supplementary Material 9

Supplementary Material 10

Supplementary Material 11

## Data Availability

The scRNA-Seq processed files are available in supplementary material, while original files are uploaded to GEO under the accession no. GSE244653. Mass spectrometry (MS) data and cytokine secretion profiles are also provided as supplementary material. Additionally, cytokine secretion profiles, MS, ATAC-Seq and scRNA-seq datasets are available as a searchable platform on: https://sherlab.shinyapps.io/IPSC-derived-Microglia/. ROSMAP data can be requested at https://www.radc.rush.edu

## Abbreviations

ATP: Adenosine 5′-triphospshate
AD: Alzheimer’s disease
ADVP: Alzheimer’s Disease Variant Portal
Aβ: Amyloid beta
ACM: Astrocyte-conditioned media
CNS: Central nervous system
ATAC-Seq: Chromatin accessibility profiles
CRISPR: Clustered Regularly Interspaced Short Palindromic Repeats
CR: Complement system
CRISPRa: CRISPR activation
CRISPRi: CRISPR-interference
diaPASEF: Data-independent acquisition
DLPFC: Dorsolateral prefrontal cortex
EB: Embryoid bodies
GO: Gene ontology
HTOs: Hash-tag oligonucleotides
hiPSCs: Human induced pluripotent stem cells
HMC3: Human microglia clone 3
iMG: iPSC-derived microglia
LPS: Lipopolysaccharide
LC-MS/MS: Liquid chromatography with tandem mass spectrometry
NGS: Next Generation Sequencing
PASEF: Parallel accumulation-serial fragmentation
pMG: Primary microglia
PCA: Principal component analysis
sgRNA: Single guide RNA
SNV: Single nucleotide variants
scRNA-Seq: Single-cell RNA sequencing
Tet-CRISPR: Tetracycline-regulatable CRISPR
TRE: Tetracycline-responsive
FDR: False discovery rate
VLMC: Vascular and leptomeningeal cells

## Code Availability

All analysis Python code is available as a GitHub repository (https://github.com/jasoncngo/IPSC-Derived-Microglia).
