# Supplementary material for: An integrated toolkit for human microglia functional genomics
Source: Stem Cell Res Ther. 2024 Apr 10;15:104. doi: 10.1186/s13287-024-03700-9 (PMC11005142; doi:10.1186/s13287-024-03700-9)
Supplement: Supplementary file 9 — Supplementary Material 9 [file 13287_2024_3700_MOESM9_ESM.docx]

**An integrated toolkit for human microglia functional genomics**

Imdadul Haq^1,2,3^, Jason C. Ngo^1,2,3^, Nainika Roy^1,2,3^, Richard L. Pan^1,2,3,8^, Nawsheen Nadiya^1,2,3^, Rebecca Chiu^1,2,3,7^, Ya Zhang^1,2,3,7^, Masashi Fujita^1,2,3,7^, Rajesh K. Soni^6^, Xuebing Wu^4^, David A Bennett^5^, Vilas Menon^1,2,3^, Marta Olah^2,3^, and Falak Sher*^1,2,3^

Author affiliations

^1^Center for Translational and Computational Neuroimmunology, Columbia University Medical Center, New York, NY USA

^2^Taub Institute for Research on Alzheimer’s Disease and Aging Brain, Columbia University Medical Center, New York, NY USA

^3^Department of Neurology, Columbia University Medical Center, New York, NY USA

^4^Department of Medicine and Department of Systems Biology, Columbia University Irving Medical Center, New York, NY 10032, USA

^5^Rush Alzheimer’s Disease Center, Rush University Medical Center, Chicago, IL USA

^6^Proteomics Core, Department of Pathology and Cell Biology, Columbia University Medical Center, New York, NY USA

^7^Neuroimmunology Core, Center for Translational & Computational Neuroimmunology, Division of Neuroimmunology, Department of Neurology, Columbia University Medical Center, New York, NY USA

^8^Department of Physiology and Cellular Biophysics, Columbia University Medical Center

*Corresponding author: [fs2644@cumc.columbia.edu](mailto:fs2644@cumc.columbia.edu)

**Step-by-step iMG differentiation protocol**

1. **Biological materials**

Human iPSCs derived from CD34^+^ cord blood cells from a female, using an EBNA-based episomal system, was purchased from Gibco (A18945). All the relevant institutional and governmental regulations regarding the use of iPSCs were followed.

**B. Reagents**

**! CAUTION** All the necessary precautions should be taken during reagent handling. The BSL-1 and BSL-2 lab safety protocols should be followed during non-tissue culture work and tissue culture work, respectively. For unfamiliar reagents, specific instructions on reagent material safety data sheets should be followed.

**Growth medium and supplements for iPSCs**

**Δ Critical** We have not used growth medium and supplements from any other vendors.

- mTeSR^TM^1 (StemCell Technologies, Cat. No. 85850)

**Note:** We have also used mTeSR^TM^ plus (StemCell Technologies, Cat. No. 100-0276) to maintain cells and we did not observe any difference.

- ReLeSR^TM^ (STEMCELL Technologies, Cat. No. 05872)
- RHO/ROCK pathway inhibitor, Y-27632 (Dihydrochloride) (StemCell Technologies, Cat. No. 72304)
- Geltrex Ldev free RGF BME (Life Tech Corp, Cat. No. A1413202)

**Box 1 | | iPSC maintenance and culture**

**Note:** If thawing cells for the first time, thaw according to the manufacturer’s instructions and then proceed using the following methods. After thawing, wait until the cells are roughly 70-80% confluent before passaging. At this point, there should be roughly 3.5-4 million cells in a 6-well plate. The following instructions can be used for the first passage after thawing and subsequent maintenance.

1. Coat a 6-well tissue culture treated plate with 1mL Geltrex for one hour at 37°C in the CO_2_ growth incubator.

**Note:** Add 1mL of Geltrex at a final concentration of 1% to serum-free, ice-cold IMDM media (cold media is required for proper dissolution) and then add to the plate. Keep the plate at 37°C for an hour and then wash with PBS three times. Add 1.5mL mTeSR^TM^ media supplemented with 10μM ROCK inhibitor to the plate and prewarm at 37°C for 10 minutes in CO_2_ incubator.

1. Seed 250K cells in one well of a 6-well plate with 1.5mL mTeSR^TM^ media supplemented with 10μM ROCK inhibitor.
2. Replace the media next day with 1.5mL mTeSR^TM^ media supplemented with 5μM ROCK inhibitor.
3. After 48 hours, replace the media next day with 1.5mL mTeSR^TM^ media without any ROCK inhibitor.
4. After this, replace the media every other day with 1.5mL mTeSR^TM^ media without any ROCK inhibitor until the next passage point.
5. Passage the cells when they reach 70% confluency.

**Note:** For passaging, remove the mTeSR^TM^ media from the cell and wash with 1mL PBS once. Add 1ml of ReLeSR^TM^ to the cells (for one 6-well plate). Incubate for 1minute at 37°C in the CO_2_ growth incubator. Remove 800μl of ReLeSR^TM^ from the cells and incubate for two more minutes. After incubation, remove the rest of ReLeSR^TM^ and add 1ml of mTeSR^TM^ media supplemented with 10μM ROCK inhibitor to resuspend the cells. Count the cells and seed on the Geltrex-coated plate.

**Growth medium and supplements for differentiating iPSC**

- STEMdiff Hematopoetic Kit (StemCell Technologies, Cat. No. 05310)
- RHO/ROCK pathway inhibitor, Y-27632 (Dihydrochloride) (StemCell Technologies, Cat. No. 72304)
- Geltrex Ldev free RGF BME (Life Tech Corp, Cat. No. A1413202)

**Growth Medium, growth factors, and supplements for Microglia Progenitor Cells (MPC)**

- IMDM (Gibco Cat. No. 31980-030)
- Defined FBS (Lonza, Cat. No. CC-4123)
- Penicillin-Streptomycin (Gibco, Cat. No. 15140122)
- Geltrex Ldev free RGF BME (Life Tech Corp, Cat. No. A1413202)
- Poly-L-lysine (Sigma, Cat. No. P4707-50ML)
- M-CSF (Preprotech, Cat. No. 300-25)
- CM-CSF (Preprotec, Cat. No. 300-03)
- IL3 (Preprotech, Cat. No. 200-03)

**Growth medium and supplements for Astrocytes**

- ABM basal medium (Lonza, Cat. No. CC-3187)
- HEPES Buffer Saline Solution (HBSS) (Lonza, Cat. No. CC-5024)
- Trypsin/EDTA (Lonza, Cat. No. CC-5012)
- Trypsin Neutralization Solution (TNS) (Lonza, Cat. No. CC-5002)
- Poly-L-lysine (PLL) (Sigma, Cat. No. P4707-50ML)

**Box 2 ⏐⏐ Astrocyte maintenance and culture**

**Note:** If thawing cells for the first time, thaw according to the manufacturer’s instructions and then proceed using the following methods. After thawing, wait until the cells are roughly 70-80% confluent before passaging. At this point, there should be roughly 1.5-2 million cells in a 6-well plate. The following instructions can be used for the first passage after thawing and subsequent maintenance.

1. Coat a 6-well culture plate with 1mL PLL for an hour in the 37°C growth chamber before seeding the cells.
2. After coating the plate, remove the PLL, wash twice with 1mL PBS and add 1.2mL of ABM medium and keep in the 37°C growth chamber.
3. Wash the plate with the cells twice with 1mL HBSS, add 1mL Trypsin/EDTA to the cells and keep at 37°C for 4 minutes.
4. Take the plate out and tap the bottom of the well lightly to help detach any still detached cells. Check under the microscope for detachment; 90% of the cells should be detached. If not detached fully, keep the cells at 37°C for one additional minute.
5. Take out the plate and add 1mL TNS to the well (the volume of TNS should be equal to the volume of Trypsin/EDTA in the well); mix by gentle pipetting.
6. Transfer the cells to a 15mL conical tube.
7. Wash the well with 1mL of HBSS to collect the rest of the cells and add to the same tube.
8. Centrifuge at 250g for 3 minutes using a swinging bucket centrifuge.
9. Discard the supernatant.
10. Resuspend the cells in 0.3mL ABM medium and add to the PLL coated plate; the final media volume should be 1.5mL.
11. For regular maintenance, seed 300K cells in one well of a 6-well culture plate. If the conditioned medium from the astrocyte cells is needed, seed two separate sets of the astrocyte culture; use one for maintenance and one for preparation of the conditioned medium.

**Note:** Aliquot HBSS, TNS, and Trypsin/EDTA and keep at -20°C. Before use, thaw at 4°C and warm up at 37°C right before use.

**Δ Δ Δ Important: Composition of Conditioned MPC Medium (CMM)**

Growth factors for MPC medium are IL-3, GM-CSF, and M-CSF with a final concentration of 20 ng/ml.

- MPC medium: IMDM supplement with 10% defined FBS and 1% Penicillin-Streptomycin.

**Note:** MPC medium is good for 2 weeks at 4°C. Add growth factors to the medium right before use.

- Conditioned MPC Medium: Half of the MPC Medium, half of the supernatant from the astrocyte culture media, and growth factors.
- Grow astrocytes in ABM basal medium to 70% confluency using the protocol above.
- Replace the ABM medium with 1.5mL MPC medium supplemented with IL-3, GM-CSF, and M-CSF with a final concentration of 20 ng/ml.
- After the addition of the MPC medium, culture the astrocytes for 48 hours before collecting the medium.
- Filter the medium using an 0.45 μm syringe filter.
- Mix the filtered astrocytic medium with fresh MPC medium at a 1:1 ratio.
- Add fresh growth factors; IL-3, GM-CSF, and M-CSF with a final concentration of 20 ng/ml

**Note:** Astrocyte cultures with MPC medium can be used for up to two weeks for supernatant collection. Media can be collected every 48 hours and should be replaced with 1.5mL MPC medium supplemented with freshly added growth factors.

**Growth medium, growth factors, and supplement for iMG**

**Δ Critical** We have not used growth medium and supplements from any other vendors.

- Neurobasal Medium (ThermoFisher Scientific, Cat. No. 21103049)
- Sodium L-lactate (Sigma, Cat. No. L7022-5G)
- L-Ascorbic Acid 2-phosphate sesquimagnesium salt hydrate (Sigma, Cat. No. A8960-5G)
- AlbuMAX I Lipid-Rich BSA (ThermoFisher Scientific, Cat. No. 11020021)
- N2 NeuroPlex^TM^ Serum-Free Supplement (Gemini Biosciences, Cat. No. 400-163)
- Gem21 NeuroPlex^TM^ without Vitamin A Serum-Free Supplement (Gemini Biosciences, Cat. No. 400-161)
- DL-Lactic Acid, 85+% (ThermoFisher Scientific, Cat. No. 412965000)
- Sodium Pyruvate (Gibco, Cat. No. 11360070)
- Glutamax (Lonza, Cat. No. CC-4123)
- Biotin (Sigma Cat. No. B4639)
- Sodium Chloride (Invitrogen, Cat. No. AM9760G)
- Penicillin-Streptomycin (Gibco, Cat. No. 15140122)
- Poly-L-lysine (Sigma, Cat. No. P4707-50ML)
- CM-CSF (Preprotec, Cat. No. 300-03)
- IL34 (Preprotec, Cat. No. 200-34)

**Growth medium composition for iMG**

- Neurobasal Media (250 ml), Gem21 (10 ml), N2 (5 ml), Lactic Acid 85% stock (100 ul), 100mM Sodium Pyruvate (5 ml), Glutamax (5 ml), Biotin (1.75 mg), Albumax Lipidated BSA (1 g), Ascorbic Acid (1.25 mg), Penicillin-streptomycin (5 ml), 5M NaCl (5 ml). All the reagents were added together and filter sterilized. M-CSF and IL-34 were added to a final concentration of 20 ng/ml before using on iMG cells.

**Note:** Aliquot the media and keep at -20°C. The media is good for 6 months at -20°C. Before use, thaw at 4°C. The media is good for up to a week at 4°C. Add growth factors to the medium right before use for cell culture and warm up at 37°C.

**Reagents and materials for functional assays**

- RNeasy Mini Kit (QIAGEN, Cat. No. 74104)
- iScript^TM^ cDNA Synthesis Kit (Biorad, Cat. No. 1708891)
- SYBR Green (ThermoFisher, Cat. No. 4385612)
- RIPA Buffer (Cell Signaling Technology, (Cat. no. 9806S)
- BCA protein assay kit (ThermoFisher, Cat. no. 23228)
- Dithiothreitol, DTT (Sigma, Cat. No. 1019777001)
- 3X Red Loading Buffer (Cell Signaling Technology, Cat. No. 67068S)
- 10 Tris/Glycine/SDS buffer (Bio-Rad, Cat no. 1610732)
- 4-20% Mini-Protean TGX Precast Protein Gels, 10 well, 50 μl (Bio-Rad, Cat. No 4561094)
- Tris-Buffer Saline 10X, pH 7.4 (Boston BioProducts, Inc, Cat. No. BM-300-LT)
- 20X TBS Tween^TM^-20 (Thermo Fisher, Cat. No. 28360)
- Intercept Blocking Buffer (LI-COR, Part No. 927-60001)
- Phosphate-buffered saline (ThermoFisher, Cat. No. J61196.AP)
- Poly-L-lysine (Sigma, Cat. No. P4707-50ML)
- Blasticidin (Invitrogen, Cat. No. Ant-bl-05)
- Puromycin dihydrochloride (Sigma-Aldrich, Cat. No. P8833)
- eBioscience^TM^ Lipopolysaccharide (LPS) Solution (Invitrogen, Cat. No. 00-4976-03)
- Doxycycline Hyclate (Sigma, Cat. No. D9891)
- beta-Amyloid (1-42), HiLyteTM Fluor 647-labeled – 0.1 mg (ANASPEC, Cat. No. AS-65161)
- Millex-HV Syringe Filter Unit, 0.45 μm (MilliporeSigma, Cat. No. SLHVR33RS)
- BD Syringes without Needle, 50 mL (BD Bioscience, Cat. No. BD 309654)
- Corning^TM^ Falcon^TM^ Round-Bottom Polystyrene Test Tube with Cell Strainer Snap Cap, 5 ml (Corning, Cat no. 352235)
- 38.5 ml, Open-Top Thinwall Ultra-Clear Tube, 25 X 89 mm (Backman Coulter, Cat No. 344058)

1. **Antibody index**

| Name | Company (Cat. No.) | Host | Dilution | Purpose |
| --- | --- | --- | --- | --- |
| CD11b | BioLegend (101217) Alexa Fluor 488 |  | 1:100 | Flow Cytometry |
| CD34 | BioLegend (343503) FITC |  | 1:100 | Flow Cytometry |
| CD43 | BioLegend (315203) FITC |  | 1:100 | Flow Cytometry |
| CD45 | BioLegend (103112) APC |  | 1:100 | Flow Cytometry |
| IBA1 | Wako (NC1801858) | Goat | 1:100 | ICC |
| TEEM119 | Abcam (ab185333) | Rabbit | 1:100 | ICC |
| SORL1 | CST (79322S) | Rabbit | 1:3000 | Western Blot |
| GAPDH | CST (2118S) | Rabbit | 1:4000 | Western Blot |
| Cas9 | Abcam (ab191684) | Mouse | 1:2000 | Western Blot |

**D. Primers and sgRNA Index**

| Gene | Forward Primer/sgRNA | Reverse Primer | Purpose |
| --- | --- | --- | --- |
| *GAPDH* | ACAACTTTGGTATCGTGGAAGG | GCCATCACGCCACAGTTTC | qPCR |
| *C1QA* | TCTGCACTGTACCCGGCTA | CCCTGGTAAATGTGACCCTTTT | qPCR |
| *C1QB* | ATCAACTCCCAATTTCGTGGTT | GGTGGTCATATAAGGCCCAGT | qPCR |
| *C1QC* | CCAACCCGCAGGGAGATTATG | CCGAGTTGACCTGATTGGTTTT | qPCR |
| *ITGAM* | GCCTTGACCTTATGTCATGGG | CCTGTGCTGTAGTCGCACT | qPCR |
| *P2RY12* | CACTGCTCTACACTGTCCTGT | AGTGGTCCTGTTCCCAGTTTG | qPCR |
| *CD33* | GGTGTGACTACGGAGAGAACC | GGTAGGGTGGGTGTCATTCC | qPCR |
| *CD68* | GGAAATGCCACGGTTCATCCA | TGGGGTTCAGTACAGAGATGC | qPCR |
| *AIF1* | AAGAAGTGGTCTGACCTCAAGA | AGGTTGCAGATACGTTGTTGC | qPCR |
| *SORL1* | CAAGGTGTACGGACAGGTTAGT | CCAATGCCAGGCTATCTCG | qPCR |
| *TREM2* | CATCACAGACGATACCCTGGG | GCAGATGGGAGCCTTGAGAT | qPCR |
| sgRNA5 (i5) | CAGTAGCGTTCGCCCGAACA |  | LV |
| sgRNA2 (a2) | TGCTCCGTGTCGCCATGTTC |  | LV |

**E. Protocol for iPSC derived microglia (iMG)**

**Step 1: Differentiation if iPSCs into hematopoietic progenitor cells**

1. Use 12 or 6 well plates. Note that the following volumes are for 6 well plates and can be scaled down or up as necessary.
2. Coat plate with 1mL Geltrex for 1 hour.

**Note:** Add 1mL of Geltrex at a final concentration of 1% to serum-free, ice-cold IMDM media (cold media is required for proper dissolution) and then add to the plate. Keep the plate at 37°C for an hour and then wash with PBS three times. Add 1.5mL mTeSR^TM^ media supplemented with 10μM ROCK inhibitor to the plate and prewarm at 37°C for 10 minutes in CO_2_ incubator.

1. Seed 50K iPSCs in 1.5mL mTeSR^TM^ media supplemented with ROCK inhibitor (10μm in final concentration).
2. Replace the media the next day with 1.5mL STEMdiff Hematopoietic basal medium (StemCell, Cat 05311) supplemented with reagent A. This is Day 1.
3. On day 3 (after 48 hours), replace half of the media (750uL) with fresh STEMdiff Hematopoietic basal medium supplemented with reagent A.
4. On day 4, replace all of the media with 1.5mL STEMdiff Hematopoietic basal medium supplemented with reagent B.
5. On day 6, replace half of the media (750uL) with fresh STEMdiff Hematopoietic basal medium supplemented with reagent B.
6. On day 8, replace half of the media (750uL) with fresh STEMdiff Hematopoietic basal medium supplemented with reagent B.
7. On day 10, replace half of the media (750uL) with fresh STEMdiff Hematopoietic basal medium supplemented with reagent B.
8. On day 12, detach cells and seed in Poly-L-Lysine (PLL) coated 6 well plates.

At this stage the cells are hematopoietic progenitors and can be cryopreserved for future differentiations if required.

**Cryopreservation protocol for hematopoietic progenitor cells (HPCs**)

i. Preparation for Freezing:

- Centrifuge the required number of HPCs at 300g for 5 minutes.

- Remove the medium and gently resuspend the cells in a calculated volume of HPCs freezing medium (refer to the recipe below).

*Note: Typically, we freeze HPCs in cryovials at a density of 1 million cells/ml using the HPCs freezing medium*.

ii. Freezing Process: Promptly transfer the cell suspension into cryovials. Place the cryovials in a Mr. Frosty/Cell Camper and store at -80°C.

iii. Long-term Storage: After 24 hours at -80°C, transfer the cryovials to a cryotank for storage. Important: *Prolonged storage of HPCs at -80°C beyond 24 hours can significantly reduce cell viability.*

Recipe for HPCs Freezing Medium: KOSR: 90% Sterile DMSO: 10%

Example: To prepare 50ml of HPCs freezing medium, mix 45ml of KOSR with 5ml of DMSO.

**Step 2: Maturation of HPCs into microglia**

**Note:** Add 1mL of PLL in one well of a 6-well plate for one hour in the 37°C growth chamber. After one hour, remove the PLL and wash with 1mL PBS three times. Add 1.2mL of conditioned MPC medium supplemented with IL-3, GM-CSF, and M-CSF with a final concentration of 20 ng/ml to the coated plate and keep at 37°C in the CO2 growth chamber for 20 minutes before use.

**Note:** To detach cells, remove the media and wash with 1mL PBS. Then add 1mL of Accutase and incubate in the 37°C growth chamber for 5 minutes. After incubation, neutralize by adding 1mL of the MPC medium. Homogenize the detached cells by pipetting three times and then filter through a Cell-Strainer Cap and centrifuge at 120g for 3 min. Discard the supernatant and resuspend cells with 0.3mL of conditioned MPC medium supplemented with IL-3, GM-CSF, and M-CSF with a final concentration of 20 ng/ml and add to the prepared 6-well plate. The cells should be in a final volume of 1.5mL. DO NOT remove supernatant using an aspirator.

**Δ Critical** Conditioned MPC Medium is one of the key components for the shortened iPSC-derived iMG protocol.

1. Cells seeded in a Conditioned MPC medium are considered iMG progenitor cells (MPC).

**Note:** Cells should be CD34, CD45, and CD43 positive. Flow cytometry can be used to confirm the expression of the proteins.

1. Culture cells in Conditioned MPC Medium for the next 8 days. Change the medium every two days.

**Note:** Cells can be collected for qPCR to check for marker gene expression during that period.

1. After 8 days in Conditioned MPC Medium, floating iMG can be collected.

**Note:** At day 20, iMG cells are around 25-30 μM in diameter with larger nuclei and bright outer circles when observed under bright-field microscopy. iMG at this point should express microglial markers such as TMEM, IBA1, and PU.1.

**Collecting and seeding iMG cells in a near-homeostatic state for functional assay:**

1. Collect 1.5mL of the supernatant containing the floating iMG cells.

**Note:** After collecting the floating cells, replace the media with 1.5mL conditioned MPC media supplemented with freshly added growth factors.

1. Centrifuge the floating iMG at 120g for 3 minutes.
2. Remove the supernatant carefully with a pipette.

**Note:** Do not use an aspirator to remove the supernatant.

1. Resuspend cells with iMG media and plate on PLL coated plates. Cells can now be plated according to their downstream experimental use, such as in 8-well chamber slides, 96-well plates, or 35 mm glass bottom dishes with coverslips.

| **Plate Type** | **Number of Cells to Seed** | **Volume of Media** |
| --- | --- | --- |
| 8-well chamber slide | 25-30k iMG | 200μL |
| 96 well plate | 15-20k iMG | 100μL |
| 35mM dish | 50-60k iMG | 1.25mL |

**Note:** Use 100 μl of iMG medium in a well while plating in an 8-well chamber slide. Let the cells settle for an hour and then replace the medium with 150 μl of fresh iMG medium supplemented with IL-34 and G-CSF at a final concentration of 20 ng/ml. Use a similar approach for other culture plates.

**Fact:** Differentiated cells from two wells in a 12-well plate or one well of 6-well plate provided enough material for scRNA-seq, proteomics, and multiple other functional assays. Around 80K to 100K iMG cells can be collected during each collection. Healthy iMG cells for the functional assay can be collected from day 20 to day 40.

1. After plating the floating cells, fully differentiated iMG are ready after 2-3 days for experiments.

**Generation of CRISPR-Cas system in the iPSC**

**STEP 1:** Adding dCAS9-VP64 (CRISPR-a) or dCas9-KRAB (CIRSPR-i) to the iPSCs.

| **Expression type** | **Plasmid** | **Resistance** | **Tag** |
| --- | --- | --- | --- |
| Regulatable Overexpression | dCas9-VP64 (24346702) | Blasticidin |  |
| Constitutive Overexpression | dCas9-VP64 (27661255) | Blasticidin |  |
| Regulatable Knockout | dCas9-KRAB (24346702) |  | BFP |
| Constitutive Knockout | dCas9-KRAB (23849981) |  | BFP |

1. Transduce 50K iPSCs with 5 μl of the appropriate lentivirus (instructions for preparation of concentrated lentivirus can be found below).

**Note:** Lentivirus amount may have to be titrated depending on its concentration.

- 1. For overexpression, either constitutively express the components required for CRISPRa (CRISPRa) or tetracycline (doxycycline) inducible CRISPRa (Tet-CRISPRa).
  2. For knockouts, either constitutively express the components required for CRISPR interference (CRISPRi) or tetracycline inducible CRISPR interference (Tet-CRISPRi).

1. Cells should be transduced in a 48-well, Geltrex-coated plate in 0.3mL mTeSR^TM^1 media with 10 μM of ROCK inhibitor.
2. Remove the media the next day and wash cells twice with PBS.
3. Add 0.3mL mTeSR^TM^1 media with 5 μM of ROCK inhibitor.
4. Prepare the cells for selection:
   1. For CRISPRa, add 1.5 μl/ml blasticidin the next day (48 hours after transduction), and allow selection for the next 72 hours.
   2. For CRISPRi, culture and passage cells to obtain above 10 X 10^^6^ cells and then sort for BFP positive using flow cytometry. After sorting, maintaining, and culturing the cells in a Geltrex-coated plate as mentioned in the iPSC cell maintenance section.
5. The selected cells expressing the CRISPRa/i machinery can be maintained at this point. To achieve overexpression/knockout, guide RNAs must be expressed in these cells using the methods described in STEP 2.

**Note:** Cell passaging and maintenance were performed as mentioned previously in the iPSC cell maintenance section.

**STEP 2:** Adding the guide RNA to the iPSCs expressing CRISPRa/i machinery.

1. For regular or Tet-CRISPRa/i expressing cells, transduce 50K iPSCs with 5μl of the appropriate lentivirus carrying the desired sgRNAs (instructions for preparation of concentrated lentivirus can be found below).
2. Cells should be transduced in a 48-well, Geltrex-coated plate in 0.3mL mTeSR^TM^1 media with 10 μM of ROCK inhibitor.
3. The next day, remove the media and wash the cells twice with PBS. Add 0.3mL mTeSR^TM^1 media with 5 μM of ROCK inhibitor.
4. The following day, (48 hours after transduction) remove the media and add 0.3mL fresh mTeSR^TM^1 with 0.1 μg/ml puromycin but no ROCK inhibitor.
5. Select the cells for the next 72 hours. During this time, change the media once with fresh mTeSR^TM^1 with 0.1 μg/ml puromycin.

**Δ Critical** Use one drug at a time for selection to avoid stress in the cells.

1. After selection, plate the cells for differentiation as described in the differentiation section.

**Δ Critical** Do not use either blasticidin or puromycin in the cells during differentiation.

1. For the Tet-inducible system, whenever manipulation of gene expression is required, add 2 μM final concentration of doxycycline to the differentiating cells.

**Δ Critical** Do not use either blasticidin or puromycin in the cells during differentiation.

1. For the Tet-inducible system, treat with doxycycline for at least 3 days before using cells for experimentation to enable desired gene manipulation.

**Box 3 II Concentrated lentivirus preparation**

- 1. Grow and maintain HEK293T cells in DMEM medium supplemented with 10% FBS and 1 % Penicillin-Streptomycin in 15 cm tissue culture treated plates.
  2. Transfect the cells to make virus when the plate reaches 70% confluency of the cells.
  3. For transfection, mix 20 μg of the lentiviral construct plasmid of interest with 776.6 μl of OPTI-MEM, 180 μg of linear polyethyleneimine, 13.3 μg of psPAX2, 6.7 μg of VSV-G, and incubate at room temperature for 15 minutes.
  4. After incubation, add dropwise to the HEK293T cells and return the plate to the 37°C CO2 growth chamber.
  5. The next day, replace the media with fresh DMEM medium supplemented with 10% FBS and 1% Penicillin-Streptomycin.
  6. Collect the media 48 hours after the media change (72 hours after transfection). Add fresh medium to the cells.
  7. Collect media again the following day (96 hours after transfection) and combine with the previously collected media.
  8. Centrifuge the media at 500g for 5 minutes at 4°C.
  9. Collect the supernatant in a new tube and discard the pellet.

**Note:** Add 10% bleach to the pellet while discarding the pellet. Use local safety precautions while handling lentivirus.

- 1. Filter the collected supernatant with a 0.45 μM filter using a syringe.
  2. Transfer the filtered supernatant to an ultracentrifuge tube.
  3. Centrifuge at 26,000g for 4 hours at 4°C using an ultracentrifuge.
  4. Remove the supernatant by decanting the tube.
  5. Let the tube dry for 15 minutes at room temperature.

**Note:** Lentiviral pellet should be visible at the bottom of the tube.

- 1. Add 100-200μL of serum-free medium to the pellet and keep at 4°C overnight with gentle agitation to resuspend the pellet.
  2. After overnight agitation, aliquot the lentivirus to avoid freeze/thaw cycles and keep at -80°C.
  3. Prepared lentiviruses are good for six months up to a year at -80°C.

**Testing iMG purity**

iMG differentiated using above mentioned protocol exhibit high mRNA expression of ITGAM, TREM2, C1QA, C1QC, CD33, CD68, and low expression of P2RY12 and CX3CR1 at day 20 (microglia-like floating cells). By subculturing theses floating cells in homeostatic conditions, we observe high expression of P2RY12 and CX3CR1. Additionally, these cells show no expression of neuronal, astrocytic and oligodendrocyte markers like NEUN, MAP2, GFAP, OLIG1, OLIG2, and very low or no expression of monocytic genes e.g. KLF4, ITGAL, and FCN1. We recommend employing qPCR with primer sets for these genes as a test prior to conducting downstream experiments using iMG differentiated via this protocol.

Moreover, in our mass spectrometry experiments conducted on lysates from microglia-like floating cells (day 20), we detected the microglial homeostatic marker P2RY12 protein. However, for quality control of ongoing cultures, we advise confirming the expression of TMEM119, P2RY12, and CX3CR1 proteins, and morphology after culturing the microglia-like floating cells in adherent homeostatic media conditions.

**Heterogeneity in the current iMG model**

***iMG derived from three iPSC lines****: Across the three iPSC lines we utilized for iMG differentiation, we observed high and comparable expression levels of microglial signature genes. The differentiation efficacies among these lines were also similar, as shown in Figure 1F. However, some degree of heterogeneity was noted in the expression levels of certain genes, such as CD68.*

***iMG derived from consecutive harvests from one iPSC line****: The iMG obtained from consecutive harvests of a single iPSC line demonstrated consistent gene expression and morphology. We advise against using iMG after six rounds of harvesting, as extended in vitro culturing can lead to karyotypic changes in the cells. We have verified the stable karyotype of iMG up to the sixth harvesting cycle.*

***iMG derived from independent HPC differentiations from one iPSC line:*** *The heterogeneity of iMG derived from independent HPC differentiations from a single iPSC line largely depends on the technical aspects of cryopreservation and thawing. We have noted comparable differentiation potential in iMG from independently preserved HPCs, following our standardized protocol for cryopreservation and thawing, assuming stable temperature conditions in the cryotank. Details regarding the cryopreservation and thawing of HPCs have been included in the step-by-step protocol in the section “Cryopreservation protocol for hematopoietic progenitor cells (HPCs)”*
