# Supplementary material for: An integrated toolkit for human microglia functional genomics
Source: Stem Cell Res Ther. 2024 Apr 10;15:104. doi: 10.1186/s13287-024-03700-9 (PMC11005142; doi:10.1186/s13287-024-03700-9)
Supplement: Supplementary file 10 — Supplementary Material 10 [file 13287_2024_3700_MOESM10_ESM.docx]

**Expression of IRF8 transcription factor in iPSC-derived microglia (iMG)**

**
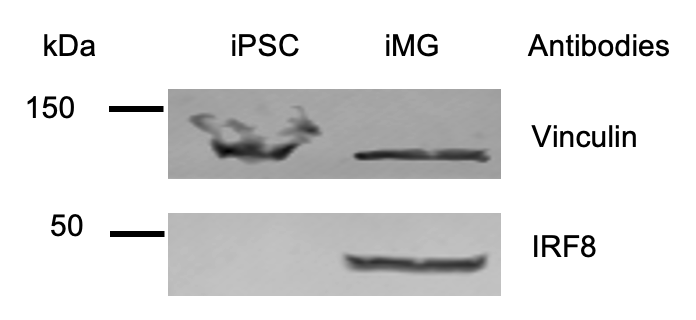
**

**Figure Ext. 1**. Western blot on lysates from iPSCs and microglial progenitors show the expression of IRF8 transcription facto

**IBA1 immunoreactivity in iMG**

In our study, as detailed in the Results section of the manuscript, we adopted a near homeostatic media approach. iPSC-derived microglia (iMG), harvested on day 20, were cultured on surfaces coated with poly-L-lysine, using homeostatic media (refer to the supplementary materials for the detailed protocol). Within 2-5 days of culture, IBA1^+^ iMG displayed the distinct morphological characteristics typical of human primary microglia cultured in vitro (Figure Ext. 2), which includes round or spindle-shaped bodies with processes, across all the induced pluripotent stem cell (iPSC) lines we tested.


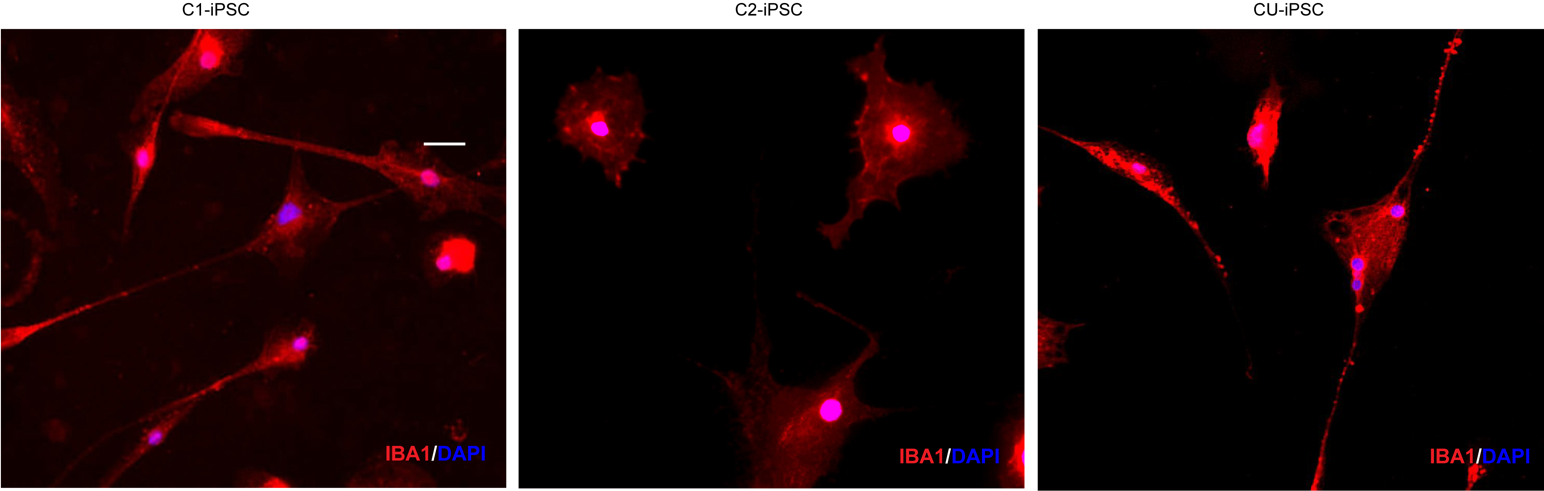


**Figure Ext.2**. **Differentiation of hiPSCs into microglia-like cells (iMG).** Immunocytochemistry (ICC) of IBA1 expression under near homeostatic conditions in iMG derived from indicated iPSC lines. Scale bars represent 20 µm.

**iMG Phagocytosis: Insight from Z-Stack confocal images**

In our results section, as presented in Figure 5A-F, we assessed the ability of our iPSC-derived microglia (iMG) to phagocytose brain-related substrates. The successful engulfment of fluorescently labeled amyloid beta by iMG was confirmed via three-dimensional analysis, utilizing a Z-stack of confocal images, as illustrated in the Extended Data Figure Ext. 3.


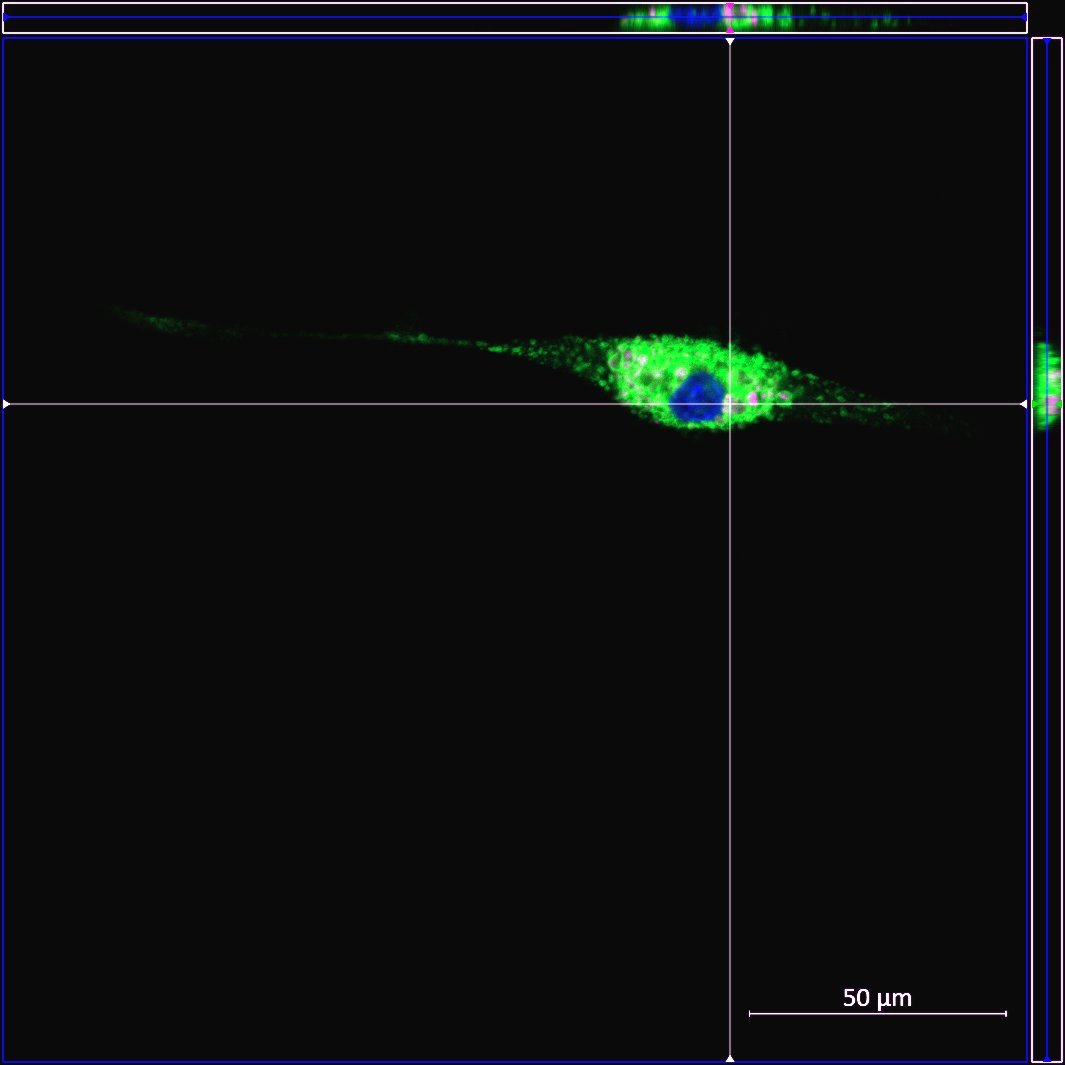


**Figure Ext. 3**. Representative confocal images of AlexaFluor647 labeled amyloid phagocytosis by iMG. Images were acquired using a Zeiss LSM 510 confocal microscope with 40X oil immersion. Cells were stained with DAPI, and anti-TMEM119 detected with AlexaFluor488 conjugated antibody. The three channels were recorded sequentially. For the Z axis, 8 planes were set up. Scale bar, 50 micrometers. Large image is a maximum intensity projection along the Z axis. Orthogonal views are included on the top and the right-hand side of the image. Cursors are centered on one representative phagosome/lysosome containing the labeled amyloid

**Intracellular Ca2+ dynamics in iMG: Response to ATP and calcimycin**

In the results section, specifically Figures 5H-J, our study focused on the intracellular calcium (Ca2+) activity in iMG in response to ATP and calcimycin (A23187), which is an ionophore commonly used for studying Ca2+ dynamics. Using quantitative image-based fluorescence analysis, we observed significant and consistent transient Ca2+ activities in iMG following exposure to both A23187 and ATP. During the ATP stimulation experiment (1µM concentration), we took interval images at 0, 150, 300, 450, and 600 seconds. For the calcimycin experiment (0.5mM concentration), images were captured at 0, 300, 600, 900, and 1145 seconds. These images effectively document the journey from initial stimulation through to the signal decay phase, with particular focus on the Ca2+ recovery process, as depicted in figure Ext. 4.


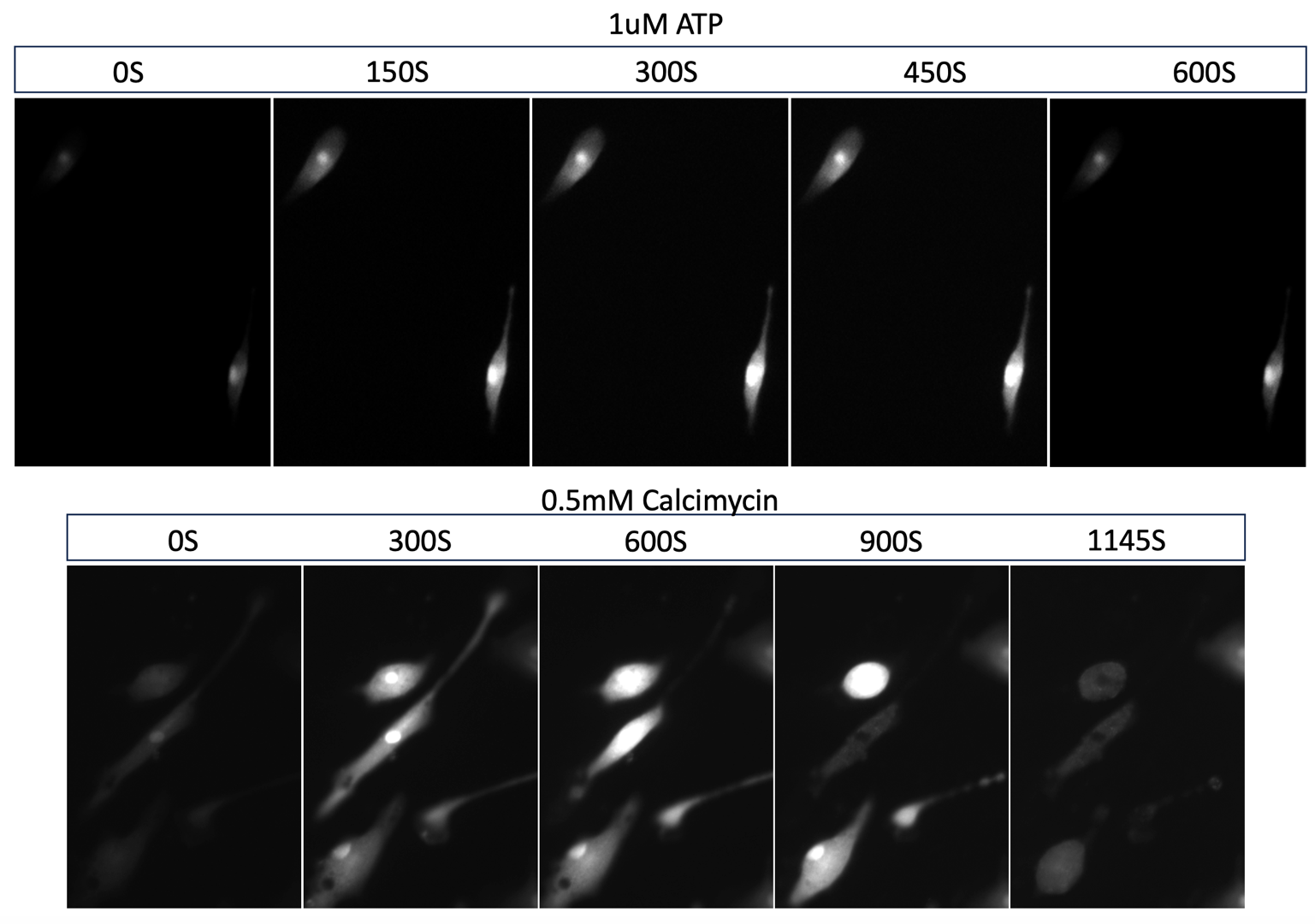


**Figure Ext. 4**. Presents two series of representative images illustrating the Fluo4 fluorescence signal in iMG. The upper panel demonstrates a calcium transient following stimulation with 1µM ATP. Initially, the baseline activity was measured, after which ATP was introduced and the activity monitored for a total duration of 600 seconds. Eventually the signal decayed back to the baseline level. The lower panel displays a selection of images showing the Fluo4 fluorescence response in iMG when stimulated with 0.5mM calcimycin. Here, the procedure started with the recording of baseline activity, followed by the addition of calcimycin, with the activity being tracked for a total of 1145 seconds, revealing a gradual decay back to baseline levels

**Calcimycin experiment calcium activity graph (0-1145 Seconds)**

Detailed graph for the calcimycin experiment, tracking the calcium activity from the start at 0 seconds to 1145 seconds, encompassing the signal decay phase.


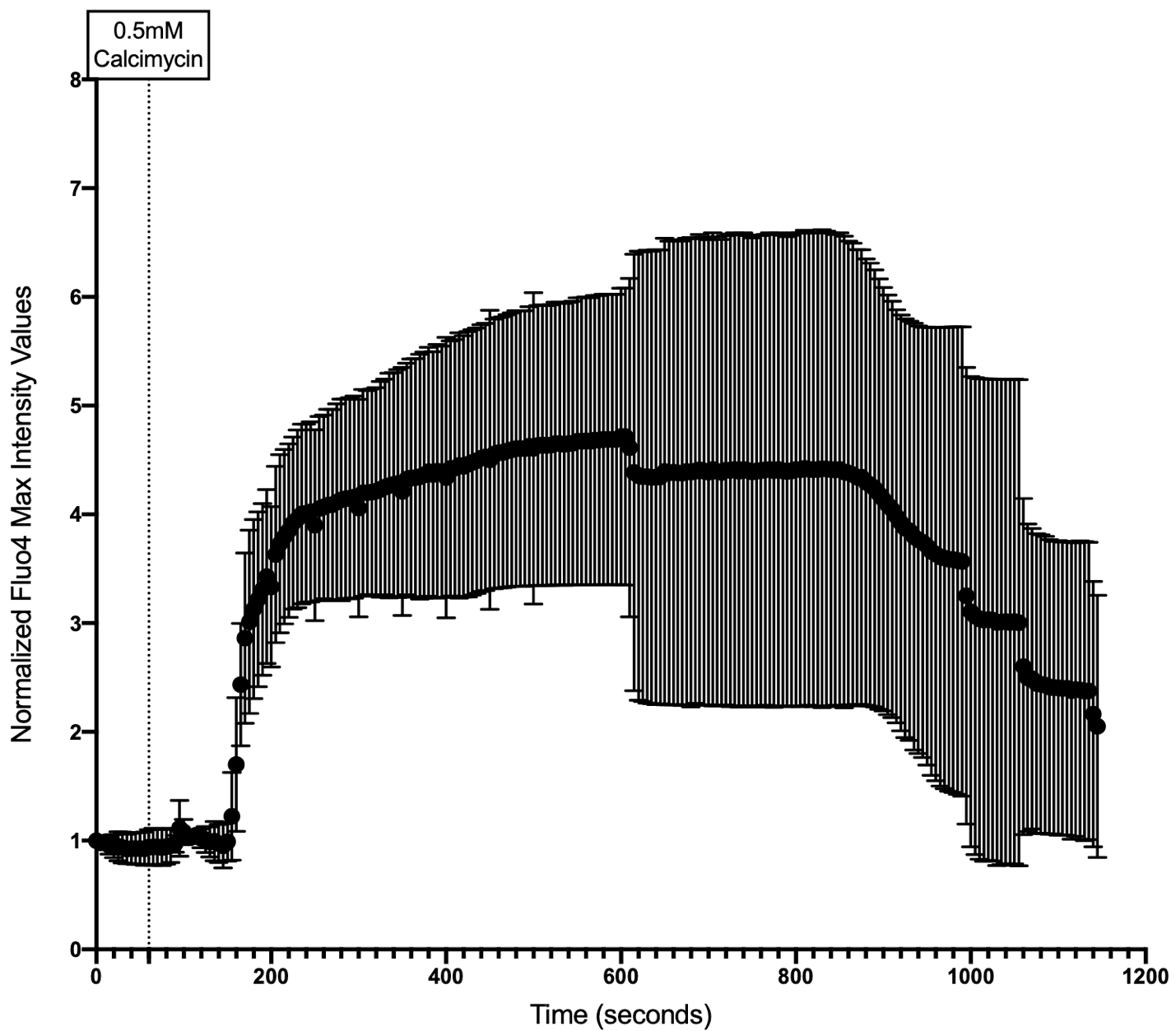


**Figure Ext. 5.** The graph displays the Fluo4 maximum intensity values observed during a calcimycin stimulation experiment over a duration of 0 to 1145 seconds. The graph clearly depicts the noticeable increase in intensity in response to the calcimycin stimulus, followed by a gradual decay of the signal towards the baseline level by 1145 seconds.
